# Associations between attainment of incentivized primary care indicators and incident sight‐threatening diabetic retinopathy in England: A population‐based historical cohort study

**DOI:** 10.1111/dom.14344

**Published:** 2021-03-03

**Authors:** Ailsa J. McKay, Laura H. Gunn, Manjula D. Nugawela, Thirunavukkarasu Sathish, Azeem Majeed, Eszter P. Vamos, German Molina, Sobha Sivaprasad

**Affiliations:** ^1^ Department of Primary Care and Public Health Imperial College London London UK; ^2^ Department of Public Health Sciences and School of Data Science University of North Carolina (UNC) at Charlotte Charlotte North Carolina USA; ^3^ Institute of Ophthalmology UCL and NIHR Moorfields Biomedical Research Centre London UK; ^4^ Centre for Population Health Sciences (CePHaS), Lee Kong Chian School of Medicine Nanyang Technological University Singapore Singapore; ^5^ Population Health Research Institute (PHRI) McMaster University Hamilton Ontario Canada; ^6^ Idalion Capital Group Charlotte North Carolina USA

**Keywords:** general practice, National Diabetes Audit, Quality and Outcomes Framework, sight‐threatening retinopathy, type 2 diabetes

## Abstract

**Aim:**

To examine the impact of attainment of primary care diabetes clinical indicators on progression to sight‐threatening diabetic retinopathy (STDR) among those with mild non‐proliferative diabetic retinopathy (NPDR).

**Materials and Methods:**

An historical cohort study of 18,978 adults (43.63% female) diagnosed with type 2 diabetes before 1 April 2010 and mild NPDR before 1 April 2011 was conducted. The data were obtained from the UK Clinical Practice Research Datalink during 2010‐2017, provided by 330 primary care practices in England. Exposures included attainment of the Quality and Outcomes Framework HbA1c (≤59 mmol/mol [≤7.5%]), blood pressure (≤140/80 mmHg) and cholesterol (≤5 mmol/L) indicators in the financial year 2010‐2011, as well as the number of National Diabetes Audit processes completed in 2010‐2011. The outcome was time to incident STDR. Nearest neighbour propensity score matching was undertaken, and univariable and multivariable Cox proportional hazards models were then fitted using the matched samples. Concordance statistics were calculated for each model.

**Results:**

A total of 1037 (5.5%) STDR diagnoses were observed over a mean follow‐up of 3.6 (SD 2.0) years. HbA1c, blood pressure and cholesterol indicator attainment were associated with lower rates of STDR (adjusted hazard ratios [95% CI] 0.64 [0.55‐0.74; *p* < .001], 0.83 [0.72‐0.94; *p* = .005] and 0.80 [0.66‐0.96; *p* = .015], respectively).

**Conclusions:**

Our findings provide support for meeting appropriate indicators for the management of type 2 diabetes in primary care to bring a range of benefits, including improved health outcomes—such as a reduction in the risk of STDR—for people with type 2 diabetes.

## 1 INTRODUCTION

Sight‐threatening diabetic retinopathy (STDR) is a common cause of preventable visual impairment in people with type 2 diabetes.^1^ Although advances in the treatment of STDR have resulted in better outcomes over the last decade,^2^, ^3^, ^4^, ^5^ preventing STDR remains the cornerstone for reducing visual morbidity.^6^, ^7^, ^8^ Optimal control of the three known modifiable risk factors, hyperglycaemia, hypertension and hyperlipidaemia, is key to preventing the development and progression of diabetic retinopathy (DR) to STDR.^6^, ^7^, ^8^, ^9^, ^10^ Clinical trials that have evaluated the role of lipid‐lowering therapies in the progression to STDR have shown that fenofibrate alone, or in combination with statins, slows the progression to STDR, but the protective effect of statins alone is less convincing.^9^, ^10^, ^11^

National screening programmes have been established in several countries worldwide to enable early identification and prompt treatment of STDR.^12^, ^13^ As screening every person with diabetes annually is challenging and not cost‐effective,^14^ various risk prediction models have been developed to predict the individual risk of STDR in order to plan risk‐based screening.^15^, ^16^ A strong predictor of STDR is the presence of DR.^15^, ^16^ The severity of DR progresses from mild, moderate or severe non‐proliferative DR (NPDR) to proliferative DR (PDR). Eyes with any severity of DR can also develop diabetic macular oedema (DMO). STDR is defined as any evidence of severe NPDR, PDR or DMO. Time to STDR differs with the severity level of DR. For example, the rate of progression to PDR in eyes with mild DR is 6.2%, while 54.8% of cases of severe NPDR progress to PDR in 1 year. Recent evidence suggests that the incidence and progression of STDR has reduced by approximately 2‐3–fold over the last 3 decades.^1^

Although the UK Prospective Diabetes Study (UKPDS) and the The Action to Control Cardiovascular Risk in Diabetes (ACCORD) study were conducted approximately 15 years apart, both trials showed that controlling systemic risk factors after the development of DR could avert or delay the onset of STDR.^6^, ^8^, ^17^ However, the impact of control of individual risk factors and their combinations on the development of STDR in people with type 2 diabetes with DR, at the population level, has not been clearly elucidated recently. The Quality and Outcomes Framework (QOF) is a pay for performance scheme that was introduced in general practice in the UK in 2004, with the aim of improving clinical outcomes for a diverse group of conditions.^18^ The framework rewards maintenance of a register of adult patients with diabetes and achieving recommended targets for control of HbA1c (≤59 mmol/mol [≤7.5%]), blood pressure (BP; ≤140/80 mmHg) and total cholesterol (≤5 mmol/L).^19^ An additional national programme, the National Diabetes Audit (NDA), compares diabetes care in England with standards set by the National Institute of Health and Care Excellence (NICE) clinical guidelines and NICE quality standards. Among other things, it monitors provision of nine care processes recommended for all patients with diabetes annually. These consist of HbA1c, BP, serum cholesterol, serum creatinine, urine albumin‐to‐creatinine ratio (ACR), foot examination, retinal screening, body mass index (BMI) and smoking review.^20^ Therefore, attainment of these QOF indicators and NDA standards is a proxy measure of optimal control of the risk factors of STDR. This study aims to provide evidence of the impact of control of risk factors on the incidence of STDR among those with mild NPDR in England.

## 2 MATERIALS AND METHODS

### 2.1 Study design and data sources

The UK Clinical Practice Research Datalink (CPRD) GOLD database, which contains longitudinal patient data collected in routine general practice and dating back to 1987, was used to obtain this retrospective cohort. Consisting of more than 18 million patients (3 million of whom are currently registered), CPRD GOLD is representative of the UK primary care‐registered population. Linked Hospital Episode Statistics (HES) and Office for National Statistics (ONS) mortality data are available for most of the CPRD participants located in England. The database has been used to investigate diabetes care processes and outcomes.^21^, ^22^ Only participants with linked HES and ONS data were eligible for inclusion. They entered the cohort on 1 April 2010 if they had an existing type 2 diabetes diagnosis, were aged 18 years or older, had been registered with their practice for at least 1 year, and were not censored prior to 1 April 2011. Only those with a mild NPDR diagnosis, as defined by the National Health Service Diabetes Eye Screening Programme (DESP) grade R1, before 1 April 2011, were included. The DESP R1 grade is equivalent to Early Treatment Diabetic Retinopathy Study (ETDRS) severity level 20‐35 inclusive and includes background microaneurysms, retinal haemorrhages with or without hard exudates and venous loops.^23^ Individuals with a type 1 diabetes or other specified non‐type 2 diabetes diagnosis occurring at any time were excluded. Those prescribed insulin within 6 months of a diagnosis made at an age younger than 35 years, or within 3 months of a diabetes diagnosis at the age of 35 years or older, were also excluded. Cohort exit occurred at the earliest of transfer out of database, last CPRD data upload, death, or 31 December 2017 (end of study period). The code lists used in deriving the cohort are available in Table S1.

### 2.2 Exposures

The exposures of interest included attainment of the QOF HbA1c (≤59 mmol/mol [≤7.5%]), BP (≤140/80 mmHg) and total cholesterol (≤5 mmol/L) indicators within the 2010‐2011 financial year. Attainment was classified according to the QOF Business Rules v38.0. Indicator status was determined using the most recent measurements in the year of interest. Where no measurement was made, the indicator was considered not to have been met. Another exposure variable describing implementation of NDA annual care processes during the 2010‐2011 year was defined by categorizing the number of completed processes as 0‐3, 4‐6 or 7‐8. NDA processes include eight measurements consisting of HbA1c, BP, cholesterol, serum creatinine, urine ACR, BMI, smoking history and foot examination. Retinal screening was not included as an exposure because study participants already had DR at baseline. All exposures were statistically defined as of the end of the baseline period (31 March 2011).

### 2.3 Outcome variable

The outcome was time to first CPRD or HES record of incident STDR (from 1 April 2011), as defined by the DESP classification.^23^ STDR included severe NPDR graded as R2, PDR (active or stable) as R3 with or without retinal photocoagulation (P1) and the presence of any sign of diabetic maculopathy recorded as M1. The grade R2 equated to ETDRS severity level 43‐57 and R3 included grades of 61 or higher.^23^

### 2.4 Covariates

The study covariates (measured at baseline before 1 April 2011) comprised sociodemographic factors (sex, age, ethnicity, 2010 patient‐level index of multiple deprivation [IMD]), the individual's primary care practice geographical region and several disease‐related variables (time from type 2 diabetes diagnosis, time from mild NPDR diagnosis, number of diabetes complications, number of glucose‐lowering therapies [GLTs] prescribed and presence/absence of insulin prescription [the latter two variables measured within 6 months before baseline]). Co‐morbidities (number of QOF registers that the individual appeared on in 2010‐2011, number of hospital admissions in the same year, and the number of prescriptions in the 6 months before cohort entry) and lifestyle factors (BMI, smoking status and alcohol use) were also included. Full variable definitions are provided in Tables S2‐S11.

### 2.5 Statistical analysis

Cohort characteristics at baseline were summarized, as well as missing data. Missing IMD patient‐level values were imputed using IMD practice‐level data. Missing lifestyle and ethnicity information was imputed from the remaining variables by employing the *mice* package in RStudio 3.5.1^24^ using five imputations. The *matchit* package was applied to conduct nearest neighbour propensity score matching using a 0.2 caliper for each exposure definition.^25^ Matched samples for each exposure were used to fit univariate and multivariate Cox proportional hazards models with the corresponding exposure included as another covariate. Concordance statistics were obtained for each of the multivariate models. Sensitivity analyses were conducted to examine the effect of QOF indicator attainment among (a) participants meeting the other two indicators (i.e. those not being considered as the exposure); (b) those not meeting either of the other two indicators; (c) those attaining lower targets such as an Hb1Ac of less than 7.0% or of even less than 6.5%, BP of less than 130/80 mmHg and cholesterol of less than 4 mmol/L; and (d) those attaining combinations of any two of HbA1c, BP and cholesterol versus attaining fewer than two indicators.

## 3 RESULTS

### 3.1 Cohort characteristics

In total, 18,978 adults (43.63% female) with type 2 diabetes diagnosed before 1 April 2010 and mild NDPR prior to 1 April 2011 were identified as eligible for inclusion, across 330 practices (see Figure S1 for a flowchart). The baseline prevalence of any DR in our cohort was 28.8%, and mild (but not sight‐threatening) retinopathy among the total who met all non‐retinopathy‐related cohort entry criteria (n = 84,441) was 22.5%. Baseline characteristics are described in Table 1. Mean (standard deviation) age was 69.5 (12.0) years, 85.7% were of White ethnic background and the average time since diabetes diagnosis was 7.9 (5.8) years. The average time since non‐sight‐threatening DR diagnosis was 4.2 (3.8) years. The majority were overweight or obese (82.8%), current or ex‐smokers (51.9%) and/or consumed alcohol (70.6%). Participants had an average of 2.4 (1.2) diabetes complications and 2.5 (1.7) co‐morbidities; and 9.2 (9.0) different prescriptions and 1.5 (1.0) different GLTs in the 6 months before study entry; also, 3803 participants (20.0%) had insulin prescriptions during that time.

**TABLE 1:** Baseline characteristics of those with a non‐sight–threatening diabetic retinopathy diagnosis (N = 18,978)

Variable	n or mean	% or SD
Age, years	69.49	11.97
Sex: female	8281	43.63%
Ethnicity		
Asian	1019	5.37%
Black	349	1.84%
Mixed	111	0.58%
Other	216	1.14%
White	16,260	85.68%
Missing	1023	5.39%
IMD		
Score	10.29	5.56%
Missing	9	0.05%
Region		
North East	524	2.76%
North West	3292	17.35%
Yorkshire and the Humber	753	3.97%
East Midlands	413	2.18%
West Midlands	2280	12.01%
East of England	1965	10.35%
South West	2586	13.63%
South Central	2187	11.52%
London	2553	13.45%
South East coast	2425	12.78%
BMI		
Underweight	134	0.71%
Ideal weight	2973	15.67%
Overweight	6469	34.09%
Obese	9249	48.74%
Missing	153	0.81%
Never smoker	9060	47.74%
Ex‐smoker	7449	39.25%
Current smoker	2394	12.61%
Smoking: missing	75	0.40%
Alcohol (units/week)		
0	3312	17.45%
1‐14	11,210	59.07%
15‐42	1817	9.57%
>42	368	1.94%
Missing	2271	11.97%
Co‐morbidities (count)	2.51	1.70
Prescriptions within preceding 6 months (count)	9.17	9.00
Hospitalizations during 2010‐2011 (count)	0.19	0.60
Duration of diabetes (years)	7.88	5.77
Duration of retinopathy (years)	4.21	3.78
Number of diabetes complications (count)	2.41	1.17
GLT prescriptions within the preceding 6 months (count)	1.48	0.99
Insulin prescription within the preceding 6 months (yes/no)	3803	20.04%

Abbreviations: BMI, body mass index; GLT, glucose‐lowering therapy; IMD, index of multiple deprivation; SD, standard deviation.

Over a mean follow‐up of 3.6 (2.0) years, 1037 (5.5%) STDR diagnoses were observed, corresponding to a diagnosis rate of 15.08 per 1000 person‐years among individuals with DR. Furthermore, 829 (5.1%), 30 (8.6%), 95 (9.3%), 9 (8.1%), 14 (6.5%) and 60 (5.9%) STDR diagnoses were observed, corresponding to diagnosis rates of 14.28, 21.69, 23.51, 20.66, 15.69 and 15.16 per 1000 person‐years for White, Black, Asian, mixed, other and missing ethnicities, respectively.

Table 2 presents the observed distribution of QOF indicator attainment and NDA process completion by the number of indicators/processes met. The HbA1c, BP and cholesterol QOF indicators were achieved by 12,010 (63.3%), 11,247 (59.3%) and 14,855 (78.3%), respectively; 6304 (33.2%) individuals met all three indicators. NDA process completion ranged from 15,288 (80.6%; urine ACR screening) to 18,397 (96.9%; BP screening). The majority (15,598, 82.2%) completed 7‐8 NDA processes; 11,898 (62.7%) completed all eight, but only 2675 (14.1%) completed 4‐6 processes. Those who did not have a measurement in 2010‐2011 were classified as not meeting the corresponding indicator, which included 856 (4.5%), 581 (3.1%) and 1575 (8.3%) for HbA1c, BP and cholesterol, respectively.

**TABLE 2:** Number (%) of individuals who met each Quality and Outcomes Framework (QOF) indicator or completed each National Diabetes Audit (NDA) care process (columns), clustered by the number of QOF indicators or NDA care processes attained (rows)

Number of indicators/care processes met		TOTAL	QOF indicator met										
			HbA1c	BP	Total cholesterol	HbA1c	BP	Total cholesterol	Serum creatinine	Urine ACR	Foot examination	BMI	Smoking history
QOF indicators	0	1105 (5.82%)	0 (0%)	0 (0%)	0 (0%)	701 (63.44%)	837 (75.75%)	549 (49.68%)	703 (63.62%)	541 (48.96%)	667 (60.36%)	706 (63.89%)	695 (62.9%)
	1	3938 (20.75%)	946 (24.02%)	918 (23.31%)	2074 (52.67%)	3589 (91.14%)	3781 (96.01%)	3276 (83.19%)	3587 (91.09%)	2881 (73.16%)	3134 (79.58%)	3437 (87.28%)	3196 (81.16%)
	2	7631 (40.21%)	4760 (62.38%)	4025 (52.75%)	6477 (84.88%)	7528 (98.65%)	7475 (97.96%)	7274 (95.32%)	7474 (97.94%)	6335 (83.02%)	6637 (86.97%)	7105 (93.11%)	6581 (86.24%)
	3	6304 (33.22%)	6304 (100%)	6304 (100%)	6304 (100%)	6304 (100%)	6304 (100%)	6304 (100%)	6268 (99.43%)	5531 (87.74%)	5671 (89.96%)	5988 (94.99%)	5635 (89.39%)
NDA care processes	0‐3	705 (3.71%)	121 (17.16%)	214 (30.35%)	62 (8.79%)	206 (29.22%)	390 (55.32%)	87 (12.34%)	186 (26.38%)	40 (5.67%)	96 (13.62%)	141 (20%)	149 (21.13%)
	4‐6	2675 (14.1%)	1454 (54.36%)	1459 (54.54%)	1574 (58.84%)	2353 (87.96%)	2484 (92.86%)	1913 (71.51%)	2284 (85.38%)	1016 (37.98%)	1192 (44.56%)	1720 (64.3%)	1353 (50.58%)
	7‐8	15,598 (82.19%)	10,435 (66.9%)	9574 (61.38%)	13,219 (84.75%)	15,563 (99.78%)	15,523 (99.52%)	15,403 (98.75%)	15,562 (99.77%)	14,232 (91.24%)	14,821 (95.02%)	15,375 (98.57%)	14,605 (93.63%)
	8	11 898 (62.69%)	8104 (68.11%)	7370 (61.94%)	10,258 (86.22%)	11,898 (100%)	11,898 (100%)	11,898 (100%)	11,898 (100%)	11,898 (100%)	11,898 (100%)	11,898 (100%)	11,898 (100%)
TOTAL		18,978 (100%)	12,010 (63.28%)	11,247 (59.26%)	14,855 (78.27%)	18,122 (95.49%)	18,397 (96.94%)	17,403 (91.7%)	18,032 (95.02%)	15,288 (80.56%)	16,109 (84.88%)	17,236 (90.82%)	16,107 (84.87%)

Abbreviations: ACR, albumin creatinine ratio; BMI, body mass index; BP, blood pressure.

Given the low counts observed for non‐White ethnicities, wide confidence intervals were obtained and no consistent associations were observed between ethnicity and STDR across exposures. Asian ethnic background, the largest minority group recorded, was significantly associated with higher risks of STDR than White ethnic background upon accounting for three of the exposures, namely, HbA1c QOF indicator achievement, achievement of all three QOF indicators, and all QOF and NDA indicator achievement. The remaining ethnic minority groups showed no significant associations across exposures except that mixed ethnicity was significantly associated with a higher risk of STDR compared with White ethnicity upon accounting for meeting 4‐6 versus 0‐3 NDA processes.

### 3.2 Associations between QOF indicator exposures and sight‐threatening retinopathy

**FIGURE 1:**
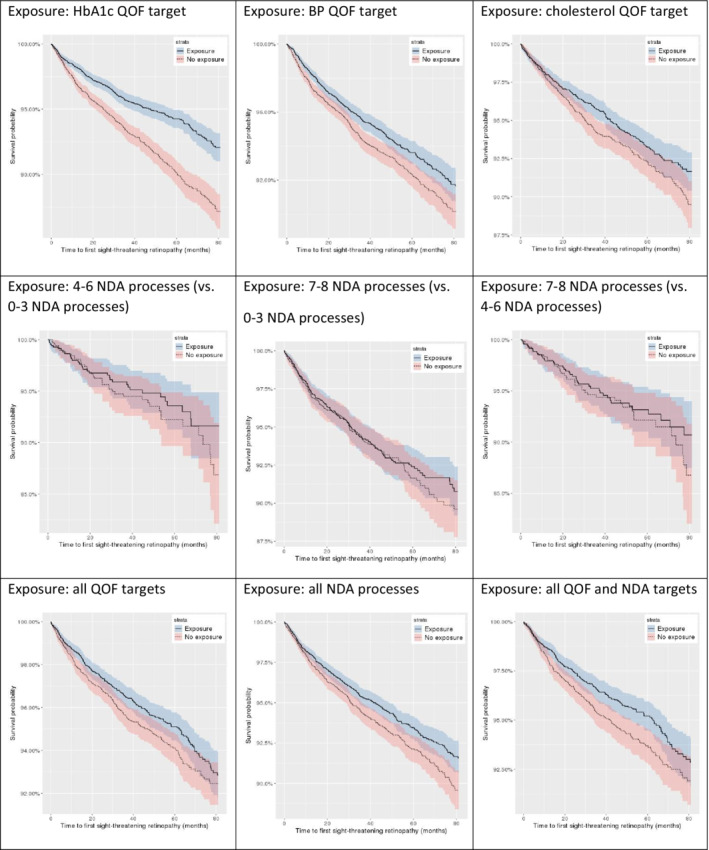
Kaplan‐Meier survival curves (and corresponding 95% CIs) for risk of sight‐threatening diabetic retinopathy after 1:1 propensity score matching across exposure definitions. BP, blood pressure; NDA, National Diabetes Audit; QOF, Quality and Outcomes Framework

Table 3 provides the unadjusted and adjusted associations between exposure to attainment of each of the QOF indicators and incident STDR. HbA1c, BP and cholesterol indicator achievement were associated with lower rates of STDR in the unadjusted and adjusted analyses (adjusted hazard ratios [HRs] [95% CI] 0.64 [0.55‐0.74; *p* < .001], 0.83 [0.72‐0.94; *p* = .005] and 0.80 [0.66‐0.96; *p* = .015], respectively). While a weak association was observed between achieving all QOF targets (vs. achieving fewer than three) and STDR in the univariate analysis, no significant association was found in the multivariate analysis (adjusted HR 0.85 [0.72‐1.01; *p* = .066]), probably as a result of simply meeting any of the QOF indicators yielding a risk reduction in STDR. Complete model results (i.e. including unadjusted and adjusted HR estimates for all covariates) are provided in Tables S2, S4, S5, and S7‐S11. Figure 1 presents Kaplan‐Meier survival curve estimates across exposure definitions.

**TABLE 3:** Unadjusted and adjusted hazard ratios (with corresponding 95% CIs and *p*‐values) for sight‐threatening diabetic retinopathy given QOF exposures after 1:1 propensity score matching

Exposure (indicator met)	Unadjusted analyses			Adjusted analyses^a^		
	HR	95% CI	*p*	HR	95% CI	*p*
HbA1c	0.60	0.52‐0.70	<.0001	0.64	0.55‐0.74	<.0001
Blood pressure	0.82	0.72‐0.94	.0033	0.83	0.72‐0.94	.0054
Cholesterol	0.82	0.68‐0.98	.0319	0.80	0.66‐0.96	.0150
All QOF indicators	0.85	0.72‐1.00	.0483	0.85	0.72‐1.01	.0659

Abbreviations: CI, confidence interval; HR, hazard ratio; QOF, Quality and Outcomes Framework.

^a^ Adjusted for age, sex, ethnicity, index of multiple deprivation, practice region, body mass index, smoking status, alcohol consumption, number of other co‐morbid conditions, hospitalizations, duration of diabetes, duration of non‐sight‐threatening retinopathy, diabetes complications, number of glucose‐lowering therapies and insulin prescription status.

### 3.3 Associations between NDA process completion exposures and sight‐threatening retinopathy

Summaries of associations between NDA care process categories and incident STDR are shown in Table 4. No association was observed among the univariate or multivariate analyses (*p* ≥ .2179) when comparing groups of the number of NDA processes met. However, the univariate analysis showed a risk reduction in STDR among those who met all eight NDA processes (unadjusted HR 0.82 [0.72‐0.95; *p* = .006]). However, this risk reduction was attenuated and became non‐significant with the multivariate analysis (adjusted HR 0.87 [0.76‐1.01; *p* = .06]). Full model results are available in Tables S3, S4, S6 and S7.

**TABLE 4:** Unadjusted and adjusted hazard ratios (with corresponding 95% CIs and *p*‐values) for sight‐threatening diabetic retinopathy given National Diabetes Audit exposures after 1:1 propensity score matching

Exposure (processes completed)	Unadjusted analyses			Adjusted analyses^a^		
	HR	95% CI	*p*	HR	95% CI	*p*
4‐6 (vs. 0‐3)	0.78	0.50‐1.22	.2772	0.75	0.47‐1.19	.2179
7‐8 (vs. 0‐3)	0.94	0.76‐1.17	.5943	0.99	0.79‐1.23	.9212
7‐8 (vs 4‐6)	0.85	0.55‐1.30	.4432	0.88	0.56‐1.37	.5649
8 (vs <8)	0.82	0.72‐0.95	.0064	0.87	0.76‐1.01	.0594

Abbreviations: CI, confidence interval; HR, hazard ratio.

^a^ Adjusted for age, sex, ethnicity, index of multiple deprivation, practice region, body mass index, smoking status, alcohol consumption, number of other co‐morbid conditions, hospitalizations, duration of diabetes, duration of non‐sight‐threatening retinopathy, diabetes complications, number of glucose‐lowering therapies and insulin prescription status.

### 3.4 Sensitivity analyses

Analyses of the QOF indicators restricted to those participants who met the other two QOF indicators showed a risk reduction in STDR only in the univariate analysis (unadjusted HR 0.78 [0.62‐0.97]; *p* = .023) (Tables S8 and S9; Figure S2). Once confounders were accounted for in the multivariate model, this association was no longer statistically significant (adjusted HR 0.83 [0.66‐1.04]; *p* = .098). A second sensitivity analysis assessed the effect of QOF indicator attainment on STDR risk among those who did not meet either of the other two indicators. Results were consistent with the primary analysis (adjusted HRs 0.45 [0.29‐0.69; *p* < .001], 0.62 [0.45‐0.85; *p* = .003] and 0.74 [0.56‐0.98; *p* = .036] for HbA1c, BP and cholesterol, respectively [Tables S10 and S11; Figure S3]). A third sensitivity analysis using lower targets such as Hb1Ac of less than 7.0% or of even less than 6.5%, BP less than 130/80 mmHg and cholesterol less than 4 mmol/L, showed that the multivariate HRs were not overly sensitive to minor changes in exposure definitions, with all 95% CIs overlapping by exposure between the primary analyses and the corresponding sensitivity analyses. Table S12, which includes HRs and 95% CIs, summarizes these comparisons, with additional details included in Tables S13 and S14 and Figure S4. The results of the fourth sensitivity analysis showed that achieving any two QOF indicators was associated with significantly better outcomes than achieving fewer than two QOF indicators (adjusted HR 0.67; 95% CI 0.57‐0.78; *p* < .0001) (Tables S15 and S16; Figure S5).

## 4 DISCUSSION

### 4.1 Summary of principal findings

Our study provides contemporary evidence of the incidence of STDR in England based on 18,978 individuals with type 2 diabetes who were previously diagnosed with DR and followed for a period of up to 6.75 years from 1 April 2011. Considering the cohort had a mean duration of diabetes of 7.9 (SD 5.8) years, 5.5% developed STDR over the study period, equivalent to 15.1 new cases per 1000 person‐years among those with mild NPDR. HbA1c, BP and cholesterol QOF indicator attainment was associated with significantly lower rates of STDR, with estimates ranging from 26% to 45%, 6% to 28% and 4% to 34% lower, respectively. Our sensitivity analyses indicate that the primary analysis results are most relevant among those who do not meet the other two QOF indicators, and hence who may have worse baseline health (i.e. those for whom meeting the QOF exposure could imply a larger marginal impact on their risk of STDR). Thus, meeting any single QOF suffices to lower an individual's risk of STDR. However, we did not explore the roles of various interventions or any other measures in achieving these QOF indicators. Therefore, further research is required in these areas. This particularly applies to the role of statins and antihypertensives in preventing the progression to STDR, where current evidence is equivocal.

### 4.2 Comparison with previous studies

The incidence of STDR observed in this study of 15.12 per 1000 person‐years among individuals with DR concurs with the declining incidence of STDR observed in other reports, despite including severe NPDR and diabetic maculopathy as an STDR event.^26^ Although the definition of progression of DR varied between studies, the ACCORD trial reported that only 12% of the study cohort had a two‐step or higher increase in severity of DR in 4 years in 2014 compared with 30% in the Wisconsin epidemiological study of diabetic retinopathy reported in 1984.^17^, ^27^ The ACCORD trial showed a stronger treatment effect of hyperglycaemia and hypercholesterolaemia in patients with mild DR than in those with no DR.^17^ The ACCORD trial and our study concur in that it is worthwhile persisting with control of hyperglycaemia and hypercholesterolaemia to reduce the rate of progression to STDR in people with DR. The ACCORD trial did not show a benefit with control of hypertension. Moreover, tight glycaemic control did not produce similar effects on DR progression in the Action in Diabetes and Vascular Disease: Preterax and Diamicron MR Controlled Evaluation (ADVANCE) trial or the Veterans Affairs Diabetes Trial (VADT).^28^, ^29^ When we compare these study cohorts, the mean age of the patients in ACCORD, ADVANCE, VADT and our study was 62, 66, 60 and 69.5 years, and the duration of diabetes was 10, 8, 11.5 and 7.88 years, respectively.^17^, ^28^, ^29^ Another point we observed was that control of any one of the risk factors in people with poor control of all three risk factors is sufficient to reduce the rates of STDR. This may explain the differences observed between VADT and ADVANCE with our study and the ACCORD trial. Although the VADT trial did not show an effect of tight glycaemic control on STDR, despite a baseline mean HbA1c of 79.2 mmol/mol (9.4%) and with 52% of the study population on insulin at baseline, the mean BP in this study population was already optimally controlled at 132/76 mmHg, and by 6 years the mean BP was 125/69 mmHg. However, our study highlights that having a BP target of 130/80 mmHg gives a 23% relative risk reduction compared with 17% for a BP target of 140/80 mmHg. Similarly, baseline HbA1c was 55.2 mmol/mol (7.2%) in the ADVANCE study compared with 65.0 mmol/mol (8.1%) and 79.2 mmol/mol (9.4%) in the ACCORD and VADT trials, respectively.^17^, ^28^, ^29^

### 4.3 Implications for policy and practice

While tight glycaemic control was defined as an HbA1c of less than 47.5 mmol/mol (6.5%) in the clinical trials, our study shows that even achieving an HbA1c of 59 mmol/mol (7.5%) is sufficient to lower the risk of progression to STDR among those with mild NPDR in whom all risk factors are suboptimally controlled. In clinical practice, it is challenging to ensure that a mean of less than 47.5 mmol/mol (6.5%) can be achieved at a population level. However, optimal control of all three risk factors is encouraged at an individual level to reduce the incidence of all complications of diabetes. Moreover, it is important that there is scope to enhance coverage of the achievement of QOF indicators, given their strong association with lower STDR incidence.

### 4.4 Strengths and limitations

Our study included a large sample, which was reasonably representative of the English population. The prevalence of any DR in the CPRD cohort was 28.8%, in keeping with the UK prevalence of 30%. Baseline prevalence of STDR was 6.3%, providing reasonable validity that our study results are generalizable. The current study adjusted for important potential confounders, and the ascertainment of exposures (routine standardized recording) was strong, with low amounts of missing data.^30^ However, certain limitations need to be acknowledged. Not all patients with DR or STDR may be coded on their medical record, leading to some underascertainment of cases. While we accounted for major confounders in the analysis, there is still a possiblity of residual confounding. While a large list of covariates were included within this study, they still comprise a closed set, and controlling for other covariates may provide further filtering of the exposure estimates. For some of the categories within covariates, there were low numbers of individuals who developed STDR, which resulted in wider confidence intervals, hence more uncertainty regarding the reliability of results for these covariates. STDR cases were comparatively few, which could have limited the statistical power for the sensitivity analysis. We did not consider the change in exposure attainment over time in the analysis, which may have potentially overestimated or underestimated the true effect estimates.

In conclusion, this study provides new information regarding the benefit of control of modifiable risk factors in reducing the incidence of STDR among those with mild NPDR at the population level. Our findings provide support for using appropriate indicators for the management of type 2 diabetes in primary care. Investing in such support for these management strategies could bring a range of benefits, including improved health outcomes—such as a reduction in the risk of STDR—for people with type 2 diabetes.

## CONFLICT OF INTEREST

The authors declare that there are no relationships or activities that might bias, or be perceived to bias, their work.

## AUTHOR CONTRIBUTIONS

LHG, AJM, EPV, AM and SS contributed to the idea generation and protocol development. AJM and LHG prepared the data for analysis and LHG performed the statistical analyses. LHG, GM, AJM, EPV, AM and SS interpreted study results. AJM, LHG and GM had primary responsibility for writing the manuscript. MN, TS, EPV, AM and SS also contributed to manuscript writing. All authors critically reviewed the manuscript. The corresponding author attests that all listed authors meet authorship criteria and that no others meeting the criteria have been omitted.

### PEER REVIEW

The peer review history for this article is available at https://publons.com/publon/10.1111/dom.14344.

## Supporting information


**Appendix S1**. Supporting Information

## Data Availability

Permission for data usage was obtained from the CPRD Independent Scientific Advisory Committee (protocol number 17_217R). Linked pseudonymised data were provided by CPRD. Data were linked by National Health Service (NHS) Digital, the statutory trusted third party for linking data, using identifiable data held only by NHS Digital. Select general practices consent to this process at a practice level with individual patients having the right to opt‐out.
