# Supplementary material for: Associations between attainment of incentivized primary care indicators and incident sight‐threatening diabetic retinopathy in England: A population‐based historical cohort study
Source: Diabetes Obes Metab. 2021 Mar 3;23(6):1322–30. doi: 10.1111/dom.14344 (PMC8350793; doi:10.1111/dom.14344)
Supplement: Supplementary file 1 — Appendix S1. Supporting Information [file DOM-23-1322-s001.docx]

**Supplementary file**

Table S1: Diabetic retinopathy code list.

| **medcode** | **Read code** | **Read term** |
| --- | --- | --- |
| 22967 | 2BBf.00 | Retinal abnormality - diabetes related |
| 11433 | 2BBP.00 | O/E - right eye background diabetic retinopathy |
| 11129 | 2BBQ.00 | O/E - left eye background diabetic retinopathy |
| 11018 | 8HBG.00 | Diabetic retinopathy 12 month review |
| 17262 | C109600 | Non-insulin-dependent diabetes mellitus with retinopathy |
| 58604 | C109611 | Type II diabetes mellitus with retinopathy |
| 42762 | C109612 | Type 2 diabetes mellitus with retinopathy |
| 18496 | C10F600 | Type 2 diabetes mellitus with retinopathy |
| 49655 | C10F611 | Type II diabetes mellitus with retinopathy |
| 1323 | F420.00 | Diabetic retinopathy |
| 7069 | F420000 | Background diabetic retinopathy |
| 10755 | F420600 | Non proliferative diabetic retinopathy |
| 11626 | F420z00 | Diabetic retinopathy NOS |
| 101881 | 2BBr.00 | Impaired vision due to diabetic retinopathy |
| 13099 | 2BBR.00 | O/E - right eye preproliferative diabetic retinopathy |
| 13103 | 2BBS.00 | O/E - left eye preproliferative diabetic retinopathy |
| 18662 | 8HBH.00 | Diabetic retinopathy 6 month review |
| 2986 | F420200 | Preproliferative diabetic retinopathy |
| 47584 | F420500 | Advanced diabetic retinal disease |
| 47328 | 2BBk.00 | O/E - right eye stable treated prolif diabetic retinopathy |
| 52041 | 2BBl.00 | O/E - left eye stable treated prolif diabetic retinopathy |
| 11599 | 7276 | Pan retinal photocoagulation for diabetes |
| 13097 | 2BBT.00 | O/E - right eye proliferative diabetic retinopathy |
| 13101 | 2BBV.00 | O/E - left eye proliferative diabetic retinopathy |
| 3286 | F420100 | Proliferative diabetic retinopathy |
| 30477 | F420700 | High risk proliferative diabetic retinopathy |
| 65463 | F420800 | High risk non proliferative diabetic retinopathy |
| 9835 | 2BBL.00 | O/E - diabetic maculopathy present both eyes |
| 52630 | 2BBo.00 | O/E - sight threatening diabetic retinopathy |
| 13102 | 2BBW.00 | O/E - right eye diabetic maculopathy |
| 13108 | 2BBX.00 | O/E - left eye diabetic maculopathy |
| 25591 | C10FQ00 | Type 2 diabetes mellitus with exudative maculopathy |
| 111798 | C10FQ11 | Type II diabetes mellitus with exudative maculopathy |
| 10099 | F420300 | Advanced diabetic maculopathy |
| 3837 | F420400 | Diabetic maculopathy |
| 19533 | 2BBY.00 | O/E - referable retinopathy |
| 39457 | F421C00 | Other intraretinal microvascular abnormality |
| 3822 | 2BB8.00 | O/E - vitreous haemorrhages |
| 25888 | 2BBm.00 | O/E - right eye clinically significant macular oedema |
| 13107 | 2BBn.00 | O/E - left eye clinically significant macular oedema |
| 100979 | 7272900 | Focal laser photocoagulation of retina |
| 33681 | 2BB..00 | O/E - retinal inspection |
| 24080 | 2BB1.00 | O/E - retina normal |
| 17198 | 2BB..11 | O/E - retina |
| 45145 | 2BB2.00 | O/E - retinal vessel narrowing |
| 48751 | 2BB3.00 | O/E - retinal A-V nipping |
| 19532 | 2BB4.00 | O/E - retinal microaneurysms |
| 8742 | 2BB5.00 | O/E - retinal haemorrhages |
| 13106 | 2BB6.00 | O/E - retinal exudates |
| 35659 | 2BB7.00 | O/E - retinal vascular prolif. |
| 3822 | 2BB8.00 | O/E - vitreous haemorrhages |
| 3914 | 2BB9.00 | O/E - retinal pigmentation |
| 36867 | 2BBa.00 | O/E- non-referable retinopathy |
| 19535 | 2BBA.00 | Examination of retina |
| 50656 | 2BBc.00 | O/E - No retinal laser photocoagulation scars |
| 31088 | 2BBD.00 | O/E - Right retina normal |
| 70163 | 2BBe.00 | O/E - right retina partially assessable |
| 31089 | 2BBE.00 | O/E - Left retina normal |
| 66273 | 2BBg.00 | O/E - right retina fully assessable |
| 26538 | 2BBi.00 | O/E - right eye no maculopathy |
| 19149 | 2BBI.00 | O/E - no retinopathy |
| 43501 | 2BBj.00 | O/E - left eye no maculopathy |
| 13100 | 2BBJ.00 | O/E - no right diabetic retinopathy |
| 47328 | 2BBk.00 | O/E - right eye stable treated prolif diabetic retinopathy |
| 13104 | 2BBK.00 | O/E - no left diabetic retinopathy |
| 52041 | 2BBl.00 | O/E - left eye stable treated prolif diabetic retinopathy |
| 9835 | 2BBL.00 | O/E - diabetic maculopathy present both eyes |
| 25888 | 2BBm.00 | O/E - right eye clinically significant macular oedema |
| 47144 | 2BBM.00 | O/E - diabetic maculopathy absent both eyes |
| 13107 | 2BBn.00 | O/E - left eye clinically significant macular oedema |
| 52630 | 2BBo.00 | O/E - sight threatening diabetic retinopathy |
| 18775 | 2BBO.00 | O/E - Laser photocoagulation scars |
| 11433 | 2BBP.00 | O/E - right eye background diabetic retinopathy |
| 11129 | 2BBQ.00 | O/E - left eye background diabetic retinopathy |
| 13099 | 2BBR.00 | O/E - right eye preproliferative diabetic retinopathy |
| 13103 | 2BBS.00 | O/E - left eye preproliferative diabetic retinopathy |
| 13097 | 2BBT.00 | O/E - right eye proliferative diabetic retinopathy |
| 13101 | 2BBV.00 | O/E - left eye proliferative diabetic retinopathy |
| 13102 | 2BBW.00 | O/E - right eye diabetic maculopathy |
| 13108 | 2BBX.00 | O/E - left eye diabetic maculopathy |
| 19533 | 2BBY.00 | O/E - referable retinopathy |
| 25116 | 2BBZ.00 | O/E - retinal inspection NOS |
| 878 | 3128000 | Fundoscopy normal |
| 1411 | 3128100 | Fundoscopy abnormal |
| 19531 | 3128.11 | Retinoscopy |
| 21754 | 3128200 | Dilated fundoscopy normal |
| 19534 | 3128300 | Camera fundoscopy |
| 22966 | 3128400 | Indirect fundoscopy following mydriatic |
| 13098 | 3128Z00 | Fundoscopy NOS |
| 17871 | 312E.00 | Direct fundoscopy following mydriatic |
| 64070 | 312G.00 | Indirect fundoscopy following mydriatic |
| 13105 | 58C1.00 | Retinal photography |
| 13196 | 66AD.00 | Fundoscopy - diabetic check |
| 18311 | 68A7.00 | Diabetic retinopathy screening |
| 11891 | 68A8.00 | Digital retinal screening |
| 881 | 3128 | Fundoscopy |
| 92317 | 2BBf.00 | O/E - left retina partially assessable |
| 36619 | 312F.00 | Camera fundoscopy |
| 30111 | 3129 | Eye fundus photography |
| 20991 | 312A.00 | Slit lamp examination |
| 95916 | 2BBH.00 | O/E - left retina fully assessable |

Table S2: Univariate hazard ratios (with corresponding 95% CIs and p-values) for risk of sight-threatening diabetic retinopathy by each covariate across QOF exposure definitions after 1:1 propensity score matching.

|  | **Exposure Definition** | | | | | | | | | | |
| --- | --- | --- | --- | --- | --- | --- | --- | --- | --- | --- | --- |
|  | **Achieve HbA1c QOF**  **Target** | | |  | **Achieve Blood Pressure QOF Target** | | |  | **Achieve Cholesterol QOF**  **Target** | | |
|  | **Hazard Ratio** | **95% CI** | **p** |  | **Hazard Ratio** | **95% CI** | **p** |  | **Hazard Ratio** | **95% CI** | **p** |
| ***Exposure*** | 0.60 | 0.52-0.70 | <0.0001 |  | 0.82 | 0.72-0.94 | 0.0033 |  | 0.82 | 0.68-0.98 | 0.0319 |
| ***Age*** | 0.99 | 0.98-1.00 | 0.0004 |  | 0.99 | 0.98-0.99 | <0.0001 |  | 0.99 | 0.98-0.99 | <0.0001 |
| ***Sex: Female*** | 0.98 | 0.85-1.13 | 0.7351 |  | 1.03 | 0.91-1.18 | 0.6161 |  | 0.96 | 0.80-1.15 | 0.6673 |
| ***Ethnicity: Asian*** | 1.62 | 1.30-2.03 | <0.0001 |  | 1.43 | 1.13-1.81 | 0.0027 |  | 1.57 | 1.14-2.15 | 0.0054 |
| ***Ethnicity: Black*** | 1.39 | 0.94-2.05 | 0.0993 |  | 1.43 | 0.98-2.08 | 0.0650 |  | 1.69 | 1.09-2.62 | 0.0182 |
| ***Ethnicity: Mixed*** | 1.28 | 0.57-2.85 | 0.5514 |  | 1.32 | 0.63-2.77 | 0.4675 |  | 1.86 | 0.83-4.17 | 0.1295 |
| ***Ethnicity: Other*** | 1.14 | 0.66-1.97 | 0.6458 |  | 0.95 | 0.54-1.68 | 0.8687 |  | 1.27 | 0.65-2.45 | 0.4843 |
| ***IMD*** | 1.00 | 0.99-1.01 | 0.7343 |  | 1.00 | 0.99-1.01 | 0.8533 |  | 1.00 | 0.98-1.02 | 0.8786 |
| ***North West*** | 0.86 | 0.70-1.04 | 0.1219 |  | 0.89 | 0.74-1.07 | 0.2036 |  | 0.83 | 0.65-1.07 | 0.1547 |
| ***Yorkshire & Humber*** | 1.27 | 0.91-1.76 | 0.1621 |  | 1.14 | 0.83-1.58 | 0.4087 |  | 0.90 | 0.54-1.48 | 0.6658 |
| ***East Midlands*** | 1.29 | 0.83-2.01 | 0.2651 |  | 1.25 | 0.83-1.89 | 0.2930 |  | 1.23 | 0.69-2.18 | 0.4798 |
| ***West Midlands*** | 0.93 | 0.75-1.16 | 0.5127 |  | 0.95 | 0.77-1.17 | 0.6286 |  | 0.95 | 0.72-1.26 | 0.7457 |
| ***East of England*** | 0.94 | 0.74-1.19 | 0.5847 |  | 0.91 | 0.73-1.15 | 0.4365 |  | 0.91 | 0.66-1.24 | 0.5458 |
| ***South West*** | 1.14 | 0.93-1.39 | 0.2042 |  | 1.06 | 0.88-1.28 | 0.5220 |  | 1.09 | 0.84-1.41 | 0.5350 |
| ***South Central*** | 1.03 | 0.83-1.28 | 0.7541 |  | 1.09 | 0.89-1.34 | 0.3942 |  | 1.05 | 0.79-1.38 | 0.7527 |
| ***London*** | 0.89 | 0.72-1.11 | 0.3018 |  | 0.90 | 0.74-1.10 | 0.3183 |  | 0.89 | 0.68-1.17 | 0.4022 |
| ***South East Coast*** | 0.99 | 0.80-1.23 | 0.9438 |  | 1.01 | 0.82-1.23 | 0.9595 |  | 1.08 | 0.83-1.41 | 0.5776 |
| ***BMI: Underweight*** | 0.94 | 0.76-1.16 | 0.5720 |  | 0.94 | 0.78-1.15 | 0.5609 |  | 0.87 | 0.66-1.13 | 0.2948 |
| ***BMI: Overweight*** | 1.15 | 0.99-1.33 | 0.0668 |  | 0.98 | 0.85-1.13 | 0.8278 |  | 1.13 | 0.93-1.36 | 0.2175 |
| ***BMI: Obese*** | 0.90 | 0.78-1.04 | 0.1501 |  | 1.04 | 0.91-1.19 | 0.5436 |  | 0.97 | 0.81-1.17 | 0.7632 |
| ***BMI: Missing*** | 1.70 | 0.96-3.00 | 0.0691 |  | 1.34 | 0.69-2.58 | 0.3836 |  | 1.37 | 0.57-3.30 | 0.4873 |
| ***Ex-Smoker*** | 0.82 | 0.70-0.95 | 0.0079 |  | 0.83 | 0.72-0.96 | 0.0094 |  | 0.92 | 0.76-1.11 | 0.3696 |
| ***Current Smoker*** | 0.87 | 0.70-1.08 | 0.2146 |  | 0.92 | 0.75-1.14 | 0.4575 |  | 0.79 | 0.60-1.05 | 0.1097 |
| ***Smoking: Missing*** | 0.96 | 0.36-2.58 | 0.9416 |  | 1.43 | 0.64-3.20 | 0.3790 |  | 1.06 | 0.26-4.25 | 0.9356 |
| ***Alcohol: 1-14*** | 0.90 | 0.78-1.04 | 0.1418 |  | 0.88 | 0.77-1.00 | 0.0543 |  | 0.82 | 0.69-0.99 | 0.0362 |
| ***Alcohol: 15-42*** | 1.02 | 0.80-1.31 | 0.8535 |  | 1.02 | 0.82-1.28 | 0.8429 |  | 0.96 | 0.70-1.31 | 0.7823 |
| ***Alcohol: >42*** | 0.78 | 0.43-1.41 | 0.4090 |  | 0.64 | 0.35-1.16 | 0.1439 |  | 0.60 | 0.27-1.35 | 0.2212 |
| ***Alcohol: Missing*** | 1.06 | 0.87-1.30 | 0.5498 |  | 1.16 | 0.96-1.41 | 0.1286 |  | 1.34 | 1.05-1.72 | 0.0195 |
| ***Morbidities*** | 0.97 | 0.92-1.01 | 0.1165 |  | 0.96 | 0.92-1.00 | 0.0506 |  | 0.94 | 0.89-1.00 | 0.0383 |
| ***Prescriptions*** | 1.01 | 1.00-1.02 | 0.0071 |  | 1.02 | 1.01-1.02 | <0.0001 |  | 1.02 | 1.01-1.02 | <0.0001 |
| ***Hospitalisations*** | 1.10 | 0.98-1.24 | 0.1013 |  | 1.16 | 1.05-1.29 | 0.0053 |  | 1.06 | 0.92-1.22 | 0.4300 |
| ***Duration of diabetes (years)*** | 1.01 | 1.00-1.03 | 0.0106 |  | 1.02 | 1.01-1.03 | 0.0005 |  | 1.01 | 1.00-1.03 | 0.0688 |
| ***Duration of retinopathy (years)*** | 1.00 | 0.98-1.02 | 0.8298 |  | 1.00 | 0.99-1.02 | 0.6213 |  | 1.00 | 0.98-1.02 | 0.9933 |
| ***Complications*** | 1.12 | 1.06-1.19 | 0.0001 |  | 1.12 | 1.06-1.19 | <0.0001 |  | 1.11 | 1.03-1.19 | 0.0087 |
| ***Glucose lowering therapies*** | 1.25 | 1.17-1.35 | <0.0001 |  | 1.41 | 1.32-1.50 | <0.0001 |  | 1.42 | 1.30-1.54 | <0.0001 |
| ***Insulin prescription*** | 1.88 | 1.62-2.17 | <0.0001 |  | 2.46 | 2.14-2.82 | <0.0001 |  | 2.39 | 1.98-2.88 | <0.0001 |

Study sizes across exposures after 1:1 propensity score matching are found in Supplementary Table 4, as they are the same between univariate and multivariate analyses.

Table S3: Univariate hazard ratios (with corresponding 95% CIs and p-values) for risk of sight-threatening diabetic retinopathy by each covariate across NDA exposure definitions after 1:1 propensity score matching.

|  | **Exposure Definition** | | | | | | | | | | |
| --- | --- | --- | --- | --- | --- | --- | --- | --- | --- | --- | --- |
|  | **Meet 4-6 NDA Processes (vs. Meet 0-3 NDA Processes)** | | |  | **Meet 7-8 NDA Processes (vs. Meet 0-3 NDA Processes)** | | |  | **Meet 7-8 NDA Processes (vs. Meet 4-6 NDA Processes)** | | |
|  | **Hazard Ratio** | **95% CI** | **p** |  | **Hazard Ratio** | **95% CI** | **p** |  | **Hazard Ratio** | **95% CI** | **p** |
| ***Exposure*** | 0.78 | 0.50-1.22 | 0.2772 |  | 0.94 | 0.76-1.17 | 0.5943 |  | 0.85 | 0.55-1.30 | 0.4432 |
| ***Age*** | 0.99 | 0.97-1.00 | 0.1154 |  | 0.99 | 0.98-0.99 | 0.0016 |  | 0.99 | 0.97-1.00 | 0.1739 |
| ***Sex: Female*** | 0.69 | 0.43-1.10 | 0.1158 |  | 0.92 | 0.73-1.14 | 0.4333 |  | 0.71 | 0.45-1.10 | 0.1222 |
| ***Ethnicity: Asian*** | 0.80 | 0.35-1.85 | 0.6087 |  | 1.43 | 1.01-2.03 | 0.0457 |  | 0.90 | 0.41-1.95 | 0.7855 |
| ***Ethnicity: Black*** | 0.92 | 0.23-3.74 | 0.9047 |  | 1.16 | 0.63-2.11 | 0.6364 |  | 0.84 | 0.21-3.40 | 0.8037 |
| ***Ethnicity: Mixed*** | 5.05 | 1.24-20.61 | 0.0241 |  | 1.54 | 0.58-4.14 | 0.3890 |  | 4.20 | 0.58-30.20 | 0.1544 |
| ***Ethnicity: Other*** | 2.22 | 0.81-6.08 | 0.1202 |  | 0.40 | 0.10-1.62 | 0.2011 |  | 1.18 | 0.37-3.74 | 0.7775 |
| ***IMD*** | 1.01 | 0.96-1.05 | 0.8122 |  | 1.00 | 0.98-1.02 | 0.7762 |  | 1.00 | 0.96-1.04 | 0.8587 |
| ***North West*** | 0.93 | 0.50-1.72 | 0.8133 |  | 0.71 | 0.51-0.99 | 0.0428 |  | 1.01 | 0.58-1.76 | 0.9762 |
| ***Yorkshire & Humber*** | 0.41 | 0.06-2.93 | 0.3731 |  | 0.71 | 0.37-1.38 | 0.3128 |  | 0.34 | 0.05-2.42 | 0.2793 |
| ***East Midlands*** | 0.72 | 0.10-5.15 | 0.7397 |  | 1.65 | 0.93-2.94 | 0.0874 |  | 0.59 | 0.08-4.22 | 0.5961 |
| ***West Midlands*** | 1.28 | 0.67-2.42 | 0.4542 |  | 0.90 | 0.64-1.27 | 0.5614 |  | 1.21 | 0.64-2.28 | 0.5580 |
| ***East of England*** | 0.98 | 0.47-2.04 | 0.9630 |  | 0.91 | 0.62-1.32 | 0.6061 |  | 0.98 | 0.49-1.96 | 0.9580 |
| ***South West*** | 0.56 | 0.26-1.21 | 0.1411 |  | 0.92 | 0.67-1.27 | 0.6077 |  | 0.46 | 0.20-1.06 | 0.0671 |
| ***South Central*** | 1.15 | 0.57-2.30 | 0.7008 |  | 1.58 | 1.18-2.12 | 0.0020 |  | 0.81 | 0.37-1.75 | 0.5832 |
| ***London*** | 1.10 | 0.59-2.04 | 0.7637 |  | 1.04 | 0.76-1.42 | 0.8226 |  | 1.37 | 0.80-2.33 | 0.2513 |
| ***South East Coast*** | 0.94 | 0.47-1.88 | 0.8522 |  | 0.75 | 0.51-1.08 | 0.1191 |  | 1.50 | 0.85-2.67 | 0.1632 |
| ***BMI: Underweight*** | 1.06 | 0.58-1.93 | 0.8491 |  | 1.17 | 0.88-1.56 | 0.2718 |  | 0.97 | 0.54-1.75 | 0.9189 |
| ***BMI: Overweight*** | 0.87 | 0.53-1.43 | 0.5838 |  | 0.93 | 0.74-1.18 | 0.5539 |  | 0.93 | 0.59-1.48 | 0.7737 |
| ***BMI: Obese*** | 1.07 | 0.68-1.68 | 0.7711 |  | 0.94 | 0.76-1.17 | 0.5906 |  | 1.01 | 0.66-1.55 | 0.9579 |
| ***BMI: Missing*** | 1.81 | 0.44-7.39 | 0.4065 |  | 1.51 | 0.68-3.40 | 0.3139 |  | 2.64 | 0.83-8.35 | 0.0993 |
| ***Ex-Smoker*** | 1.17 | 0.74-1.87 | 0.4970 |  | 0.86 | 0.68-1.08 | 0.1869 |  | 0.97 | 0.62-1.52 | 0.8965 |
| ***Current Smoker*** | 1.05 | 0.58-1.90 | 0.8811 |  | 0.80 | 0.56-1.13 | 0.2041 |  | 1.10 | 0.63-1.92 | 0.7408 |
| ***Smoking: Missing*** | 1.63 | 0.23-11.75 | 0.6281 |  | 1.39 | 0.52-3.73 | 0.5147 |  | 2.93 | 0.41-21.11 | 0.2859 |
| ***Alcohol: 1-14*** | 1.01 | 0.64-1.59 | 0.9602 |  | 0.92 | 0.74-1.15 | 0.4840 |  | 1.10 | 0.72-1.70 | 0.6520 |
| ***Alcohol: 15-42*** | 1.43 | 0.74-2.79 | 0.2894 |  | 0.80 | 0.53-1.23 | 0.3088 |  | 1.35 | 0.68-2.70 | 0.3945 |
| ***Alcohol: >42*** | 0.86 | 0.21-3.51 | 0.8350 |  | 0.30 | 0.08-1.22 | 0.0924 |  | 0.53 | 0.07-3.84 | 0.5328 |
| ***Alcohol: Missing*** | 0.50 | 0.23-1.08 | 0.0768 |  | 1.12 | 0.83-1.52 | 0.4627 |  | 0.56 | 0.30-1.06 | 0.0759 |
| ***Morbidities*** | 0.84 | 0.72-0.99 | 0.0357 |  | 0.92 | 0.86-0.98 | 0.0141 |  | 0.96 | 0.84-1.10 | 0.5311 |
| ***Prescriptions*** | 1.01 | 0.98-1.03 | 0.5330 |  | 1.01 | 1.00-1.02 | 0.0025 |  | 1.01 | 0.99-1.03 | 0.1830 |
| ***Hospitalisations*** | 1.15 | 0.83-1.59 | 0.4071 |  | 1.11 | 0.94-1.30 | 0.2225 |  | 1.11 | 0.80-1.54 | 0.5230 |
| ***Duration of diabetes (years)*** | 1.02 | 0.98-1.06 | 0.3837 |  | 1.00 | 0.98-1.02 | 0.7276 |  | 1.02 | 0.98-1.05 | 0.3562 |
| ***Duration of retinopathy (years)*** | 0.99 | 0.93-1.05 | 0.6287 |  | 0.99 | 0.96-1.02 | 0.6416 |  | 0.99 | 0.95-1.04 | 0.8002 |
| ***Complications*** | 1.30 | 1.08-1.56 | 0.0050 |  | 1.17 | 1.07-1.27 | 0.0005 |  | 1.29 | 1.08-1.53 | 0.0047 |
| ***Glucose lowering therapies*** | 1.50 | 1.20-1.87 | 0.0004 |  | 1.47 | 1.33-1.63 | <0.0001 |  | 1.21 | 0.98-1.49 | 0.0734 |
| ***Insulin prescription*** | 1.78 | 1.07-2.96 | 0.0275 |  | 2.22 | 1.78-2.78 | <0.0001 |  | 2.81 | 1.79-4.40 | <0.0001 |

Study sizes across exposures after 1:1 propensity score matching are found in Supplementary Table 5, as they are the same between univariate and multivariate analyses.

Table S4: Univariate hazard ratios (with corresponding 95% CIs and p-values) for risk of sight-threatening diabetic retinopathy by each covariate across NDA and QOF exposure definitions after 1:1 propensity score matching.

|  | **Exposure Definition** | | | | | | | | | | |
| --- | --- | --- | --- | --- | --- | --- | --- | --- | --- | --- | --- |
|  | **Achieve All QOF Targets** | | |  | **Meet All NDA Processes** | | |  | **Achieve All QOF & NDA Targets** | | |
|  | **Hazard Ratio** | **95% CI** | **p** |  | **Hazard Ratio** | **95% CI** | **p** |  | **Hazard Ratio** | **95% CI** | **p** |
| ***Exposure*** | 0.85 | 0.72-1.00 | 0.0483 |  | 0.82 | 0.72-0.95 | 0.0064 |  | 0.79 | 0.65-0.96 | 0.0157 |
| ***Age*** | 0.99 | 0.99-1.00 | 0.1234 |  | 0.99 | 0.98-0.99 | <0.0001 |  | 0.99 | 0.98-1.00 | 0.0309 |
| ***Sex: Female*** | 0.93 | 0.79-1.11 | 0.4239 |  | 1.01 | 0.88-1.17 | 0.8477 |  | 0.99 | 0.82-1.21 | 0.9422 |
| ***Ethnicity: Asian*** | 1.83 | 1.39-2.41 | <0.0001 |  | 1.52 | 1.20-1.91 | 0.0004 |  | 1.90 | 1.38-2.63 | 0.0001 |
| ***Ethnicity: Black*** | 1.23 | 0.68-2.24 | 0.4891 |  | 1.63 | 1.13-2.36 | 0.0098 |  | 1.40 | 0.73-2.72 | 0.3141 |
| ***Ethnicity: Mixed*** | 1.08 | 0.45-2.59 | 0.8721 |  | 1.25 | 0.60-2.64 | 0.5532 |  | 0.66 | 0.16-2.64 | 0.5538 |
| ***Ethnicity: Other*** | 0.95 | 0.45-1.99 | 0.8833 |  | 1.11 | 0.65-1.88 | 0.7020 |  | 1.35 | 0.64-2.85 | 0.4289 |
| ***IMD*** | 1.00 | 0.99-1.02 | 0.8416 |  | 1.00 | 0.99-1.01 | 0.8665 |  | 1.00 | 0.98-1.02 | 0.8853 |
| ***North West*** | 0.80 | 0.63-1.01 | 0.0629 |  | 0.90 | 0.74-1.08 | 0.2578 |  | 0.72 | 0.54-0.95 | 0.0221 |
| ***Yorkshire & Humber*** | 1.26 | 0.86-1.85 | 0.2265 |  | 1.10 | 0.78-1.56 | 0.5812 |  | 1.26 | 0.83-1.92 | 0.2770 |
| ***East Midlands*** | 1.52 | 0.95-2.43 | 0.0820 |  | 1.26 | 0.82-1.95 | 0.2888 |  | 1.81 | 1.12-2.94 | 0.0164 |
| ***West Midlands*** | 0.95 | 0.73-1.24 | 0.6984 |  | 0.93 | 0.74-1.16 | 0.4981 |  | 1.02 | 0.76-1.38 | 0.8900 |
| ***East of England*** | 1.02 | 0.78-1.34 | 0.8675 |  | 0.99 | 0.78-1.24 | 0.9092 |  | 0.92 | 0.67-1.26 | 0.5909 |
| ***South West*** | 1.23 | 0.98-1.55 | 0.0702 |  | 1.10 | 0.90-1.33 | 0.3612 |  | 1.30 | 1.01-1.67 | 0.0456 |
| ***South Central*** | 0.91 | 0.70-1.19 | 0.4990 |  | 1.01 | 0.81-1.25 | 0.9405 |  | 0.86 | 0.62-1.19 | 0.3641 |
| ***London*** | 0.93 | 0.73-1.19 | 0.5489 |  | 0.96 | 0.78-1.18 | 0.7033 |  | 1.05 | 0.80-1.38 | 0.7182 |
| ***South East Coast*** | 0.99 | 0.77-1.28 | 0.9636 |  | 0.93 | 0.75-1.16 | 0.5364 |  | 0.96 | 0.71-1.29 | 0.7767 |
| ***BMI: Underweight*** | 0.96 | 0.77-1.20 | 0.7085 |  | 0.84 | 0.68-1.04 | 0.1083 |  | 0.96 | 0.74-1.24 | 0.7587 |
| ***BMI: Overweight*** | 1.02 | 0.86-1.21 | 0.8524 |  | 1.02 | 0.88-1.18 | 0.7780 |  | 1.00 | 0.83-1.22 | 0.9669 |
| ***BMI: Obese*** | 0.99 | 0.84-1.17 | 0.9199 |  | 1.07 | 0.93-1.23 | 0.3490 |  | 1.05 | 0.87-1.28 | 0.5939 |
| ***BMI: Missing*** | 2.05 | 1.02-4.12 | 0.0442 |  | 1.40 | 0.75-2.61 | 0.2892 |  | 0.44 | 0.06-3.14 | 0.4130 |
| ***Ex-Smoker*** | 0.88 | 0.74-1.04 | 0.1375 |  | 0.81 | 0.70-0.94 | 0.0055 |  | 0.84 | 0.69-1.02 | 0.0854 |
| ***Current Smoker*** | 0.69 | 0.51-0.93 | 0.0143 |  | 0.85 | 0.68-1.06 | 0.1400 |  | 0.95 | 0.70-1.28 | 0.7155 |
| ***Smoking: Missing*** | 0.97 | 0.31-3.03 | 0.9635 |  | 4.77 | 0.67-33.89 | 0.1186 |  | 4.56 | 0.64-32.47 | 0.1297 |
| ***Alcohol: 1-14*** | 0.81 | 0.69-0.96 | 0.0145 |  | 0.83 | 0.73-0.96 | 0.0114 |  | 0.70 | 0.58-0.85 | 0.0003 |
| ***Alcohol: 15-42*** | 0.96 | 0.72-1.27 | 0.7694 |  | 0.95 | 0.74-1.21 | 0.6641 |  | 1.13 | 0.84-1.52 | 0.4158 |
| ***Alcohol: >42*** | 1.07 | 0.57-2.00 | 0.8329 |  | 0.47 | 0.22-0.99 | 0.0462 |  | 1.15 | 0.57-2.31 | 0.7018 |
| ***Alcohol: Missing*** | 1.45 | 1.14-1.83 | 0.0020 |  | 1.21 | 1.00-1.46 | 0.0515 |  | 1.49 | 1.12-1.97 | 0.0057 |
| ***Morbidities*** | 0.97 | 0.92-1.02 | 0.1819 |  | 0.95 | 0.91-1.00 | 0.0296 |  | 0.98 | 0.93-1.04 | 0.5811 |
| ***Prescriptions*** | 1.02 | 1.01-1.02 | <0.0001 |  | 1.01 | 1.01-1.02 | <0.0001 |  | 1.03 | 1.02-1.03 | <0.0001 |
| ***Hospitalisations*** | 1.17 | 1.02-1.33 | 0.0199 |  | 1.16 | 1.05-1.29 | 0.0043 |  | 1.13 | 0.94-1.35 | 0.2003 |
| ***Duration of diabetes (years)*** | 1.02 | 1.01-1.03 | 0.0015 |  | 1.02 | 1.01-1.03 | 0.0016 |  | 1.03 | 1.01-1.04 | 0.0002 |
| ***Duration of retinopathy (years)*** | 1.00 | 0.98-1.02 | 0.8321 |  | 1.00 | 0.98-1.02 | 0.7409 |  | 0.99 | 0.96-1.02 | 0.3724 |
| ***Complications*** | 1.11 | 1.03-1.19 | 0.0037 |  | 1.14 | 1.08-1.21 | <0.0001 |  | 1.16 | 1.08-1.26 | 0.0002 |
| ***Glucose lowering therapies*** | 1.40 | 1.29-1.52 | <0.0001 |  | 1.40 | 1.31-1.49 | <0.0001 |  | 1.48 | 1.34-1.62 | <0.0001 |
| ***Insulin prescription*** | 2.34 | 1.91-2.86 | <0.0001 |  | 2.33 | 2.01-2.69 | <0.0001 |  | 2.65 | 2.12-3.32 | <0.0001 |

Study sizes across exposures after 1:1 propensity score matching are found in Supplementary Table 6, as they are the same between univariate and multivariate analyses.

Table S5: Multivariate hazard ratios (corresponding 95% CIs and p-values) for risk of sight-threatening diabetic retinopathy by each covariate across QOF exposure definitions after 1:1 propensity score matching, including the adjusted study size (n) and C-statistic (also with corresponding 95% CI).

|  | **Exposure Definition** | | | | | | | | | | |
| --- | --- | --- | --- | --- | --- | --- | --- | --- | --- | --- | --- |
|  | **Achieve HbA1c QOF**  **Target** | | |  | **Achieve Blood Pressure QOF Target** | | |  | **Achieve Cholesterol QOF**  **Target** | | |
|  | **Hazard Ratio** | **95% CI** | **p** |  | **Hazard Ratio** | **95% CI** | **p** |  | **Hazard Ratio** | **95% CI** | **p** |
| ***Exposure*** | 0.64 | 0.55-0.74 | <0.0001 |  | 0.83 | 0.72-0.94 | 0.0054 |  | 0.80 | 0.66-0.96 | 0.0150 |
| ***Age*** | 0.99 | 0.98-1.00 | 0.0011 |  | 0.99 | 0.98-0.99 | 0.0001 |  | 0.99 | 0.98-0.99 | 0.0015 |
| ***Sex: Female*** | 1.11 | 0.94-1.31 | 0.2100 |  | 1.21 | 1.04-1.41 | 0.0145 |  | 1.09 | 0.89-1.35 | 0.3943 |
| ***Ethnicity: Asian*** | 1.38 | 1.08-1.76 | 0.0110 |  | 1.10 | 0.85-1.42 | 0.4656 |  | 1.13 | 0.79-1.60 | 0.5053 |
| ***Ethnicity: Black*** | 1.31 | 0.88-1.95 | 0.1792 |  | 1.20 | 0.82-1.76 | 0.3451 |  | 1.48 | 0.94-2.31 | 0.0870 |
| ***Ethnicity: Mixed*** | 1.22 | 0.54-2.73 | 0.6337 |  | 1.27 | 0.60-2.68 | 0.5312 |  | 1.96 | 0.87-4.41 | 0.1056 |
| ***Ethnicity: Other*** | 1.09 | 0.62-1.90 | 0.7607 |  | 0.89 | 0.50-1.58 | 0.6786 |  | 1.12 | 0.57-2.20 | 0.7361 |
| ***IMD*** | 1.00 | 0.99-1.01 | 0.8562 |  | 1.00 | 0.99-1.01 | 0.9274 |  | 1.00 | 0.98-1.02 | 0.8260 |
| ***North West*** | 0.58 | 0.39-0.86 | 0.0070 |  | 0.62 | 0.43-0.91 | 0.0130 |  | 0.46 | 0.29-0.72 | 0.0007 |
| ***Yorkshire & Humber*** | 0.82 | 0.51-1.32 | 0.4103 |  | 0.80 | 0.50-1.26 | 0.3276 |  | 0.48 | 0.26-0.90 | 0.0214 |
| ***East Midlands*** | 0.81 | 0.46-1.43 | 0.4751 |  | 0.83 | 0.49-1.41 | 0.4894 |  | 0.63 | 0.32-1.25 | 0.1853 |
| ***West Midlands*** | 0.63 | 0.42-0.95 | 0.0268 |  | 0.66 | 0.45-0.97 | 0.0355 |  | 0.50 | 0.31-0.80 | 0.0037 |
| ***East of England*** | 0.63 | 0.41-0.95 | 0.0289 |  | 0.65 | 0.43-0.97 | 0.0339 |  | 0.48 | 0.29-0.79 | 0.0036 |
| ***South West*** | 0.73 | 0.49-1.09 | 0.1218 |  | 0.71 | 0.49-1.04 | 0.0810 |  | 0.56 | 0.35-0.88 | 0.0130 |
| ***South Central*** | 0.67 | 0.44-1.01 | 0.0541 |  | 0.75 | 0.51-1.11 | 0.1530 |  | 0.55 | 0.34-0.89 | 0.0142 |
| ***London*** | 0.58 | 0.39-0.88 | 0.0096 |  | 0.61 | 0.42-0.90 | 0.0125 |  | 0.47 | 0.30-0.76 | 0.0016 |
| ***South East Coast*** | 0.63 | 0.42-0.95 | 0.0286 |  | 0.67 | 0.46-0.99 | 0.0426 |  | 0.55 | 0.34-0.88 | 0.0132 |
| ***BMI: Underweight*** | 3.66 | 0.51-26.28 | 0.1970 |  | 1.30 | 0.48-3.53 | 0.6063 |  | 3.00 | 0.41-21.69 | 0.2766 |
| ***BMI: Overweight*** | 4.15 | 0.58-29.58 | 0.1559 |  | 1.24 | 0.46-3.35 | 0.6650 |  | 3.41 | 0.48-24.42 | 0.2223 |
| ***BMI: Obese*** | 3.39 | 0.48-24.18 | 0.2230 |  | 1.09 | 0.40-2.92 | 0.8689 |  | 2.70 | 0.38-19.34 | 0.3232 |
| ***BMI: Missing*** | 6.41 | 0.83-49.38 | 0.0745 |  | 1.50 | 0.46-4.91 | 0.4987 |  | 4.30 | 0.50-36.97 | 0.1845 |
| ***Ex-Smoker*** | 0.80 | 0.68-0.94 | 0.0062 |  | 0.84 | 0.72-0.98 | 0.0305 |  | 0.92 | 0.75-1.13 | 0.4228 |
| ***Current Smoker*** | 0.77 | 0.61-0.97 | 0.0274 |  | 0.82 | 0.66-1.03 | 0.0838 |  | 0.73 | 0.54-0.98 | 0.0371 |
| ***Smoking: Missing*** | 0.98 | 0.36-2.63 | 0.9661 |  | 1.58 | 0.70-3.56 | 0.2678 |  | 1.25 | 0.31-5.05 | 0.7579 |
| ***Alcohol: 1-14*** | 0.91 | 0.75-1.10 | 0.3196 |  | 0.91 | 0.76-1.09 | 0.3144 |  | 0.84 | 0.66-1.08 | 0.1741 |
| ***Alcohol: 15-42*** | 1.02 | 0.75-1.37 | 0.9203 |  | 1.06 | 0.81-1.40 | 0.6691 |  | 0.94 | 0.64-1.37 | 0.7434 |
| ***Alcohol: >42*** | 0.75 | 0.40-1.39 | 0.3601 |  | 0.67 | 0.36-1.25 | 0.2107 |  | 0.61 | 0.26-1.41 | 0.2473 |
| ***Alcohol: Missing*** | 0.89 | 0.70-1.15 | 0.3869 |  | 0.96 | 0.75-1.22 | 0.7340 |  | 1.05 | 0.77-1.43 | 0.7658 |
| ***Morbidities*** | 0.92 | 0.87-0.97 | 0.0038 |  | 0.91 | 0.86-0.96 | 0.0005 |  | 0.92 | 0.85-0.99 | 0.0192 |
| ***Prescriptions*** | 1.00 | 0.99-1.01 | 0.3946 |  | 1.00 | 0.99-1.01 | 0.4269 |  | 1.00 | 0.99-1.01 | 0.7109 |
| ***Hospitalisations*** | 1.10 | 0.97-1.24 | 0.1262 |  | 1.15 | 1.03-1.28 | 0.0120 |  | 1.06 | 0.92-1.23 | 0.4168 |
| ***Duration of diabetes (years)*** | 1.01 | 1.00-1.02 | 0.1251 |  | 1.01 | 1.00-1.02 | 0.0412 |  | 1.01 | 0.99-1.02 | 0.2967 |
| ***Duration of retinopathy (years)*** | 1.00 | 0.98-1.02 | 0.8218 |  | 1.00 | 0.99-1.02 | 0.7239 |  | 1.00 | 0.98-1.02 | 0.9590 |
| ***Complications*** | 1.21 | 1.12-1.30 | <0.0001 |  | 1.21 | 1.12-1.29 | <0.0001 |  | 1.18 | 1.07-1.30 | 0.0009 |
| ***Glucose lowering therapies*** | 1.20 | 1.10-1.30 | 0.0001 |  | 1.26 | 1.17-1.37 | <0.0001 |  | 1.28 | 1.15-1.43 | <0.0001 |
| ***Insulin prescription*** | 1.66 | 1.42-1.94 | <0.0001 |  | 1.90 | 1.62-2.22 | <0.0001 |  | 1.80 | 1.46-2.23 | <0.0001 |
|  |  |  |  |  |  |  |  |  |  |  |  |
|  | **Value** | **95% CI (lower)** | **95% CI (upper)** |  | **Value** | **95% CI (lower)** | **95% CI (upper)** |  | **Value** | **95% CI (lower)** | **95% CI (upper)** |
| ***n after matching*** | 12,442 |  |  |  | 15,452 |  |  |  | 8,236 |  |  |
| ***C-statistic*** | 0.6573 | 0.6572 | 0.6575 |  | 0.6722 | 0.6720 | 0.6724 |  | 0.6756 | 0.6753 | 0.6760 |

Reference groups for categorical covariates include: white (ethnicity); North East (region); normal weight (BMI); non-smoker (smoking status); 0 units (alcohol consumption); and no insulin prescription (insulin use).

Table S6: Multivariate hazard ratios (corresponding 95% CIs and p-values) for risk of sight-threatening diabetic retinopathy by each covariate across NDA exposure definitions after 1:1 propensity score matching, including the adjusted study size (n) and C-statistic (also with corresponding 95% CI).

|  | **Exposure Definition** | | | | | | | | | | |
| --- | --- | --- | --- | --- | --- | --- | --- | --- | --- | --- | --- |
|  | **Meet 4-6 NDA Processes (vs. Meet 0-3 NDA Processes)** | | |  | **Meet 7-8 NDA Processes (vs. Meet 0-3 NDA Processes)** | | |  | **Meet 7-8 NDA Processes (vs. Meet 4-6 NDA Processes)** | | |
|  | **Hazard Ratio** | **95% CI** | **p** |  | **Hazard Ratio** | **95% CI** | **p** |  | **Hazard Ratio** | **95% CI** | **p** |
| ***Exposure*** | 0.75 | 0.47-1.19 | 0.2179 |  | 0.99 | 0.79-1.23 | 0.9212 |  | 0.88 | 0.56-1.37 | 0.5649 |
| ***Age*** | 0.98 | 0.96-1.00 | 0.0765 |  | 0.99 | 0.98-1.00 | 0.0044 |  | 0.98 | 0.96-1.00 | 0.0760 |
| ***Sex: Female*** | 1.25 | 0.73-2.14 | 0.4187 |  | 1.09 | 0.85-1.40 | 0.5147 |  | 0.94 | 0.56-1.57 | 0.8067 |
| ***Ethnicity: Asian*** | 0.67 | 0.27-1.69 | 0.4007 |  | 0.97 | 0.66-1.43 | 0.8846 |  | 0.89 | 0.38-2.10 | 0.7980 |
| ***Ethnicity: Black*** | 1.07 | 0.25-4.50 | 0.9289 |  | 0.91 | 0.49-1.67 | 0.7497 |  | 1.05 | 0.25-4.45 | 0.9481 |
| ***Ethnicity: Mixed*** | 4.69 | 1.02-21.66 | 0.0476 |  | 1.29 | 0.47-3.48 | 0.6211 |  | 3.09 | 0.40-23.88 | 0.2795 |
| ***Ethnicity: Other*** | 2.45 | 0.81-7.35 | 0.1109 |  | 0.38 | 0.09-1.52 | 0.1703 |  | 1.38 | 0.40-4.73 | 0.6065 |
| ***IMD*** | 1.01 | 0.97-1.05 | 0.7135 |  | 1.00 | 0.98-1.02 | 0.8244 |  | 1.00 | 0.96-1.05 | 0.9075 |
| ***North West*** | 0.38 | 0.14-1.06 | 0.0645 |  | 0.35 | 0.21-0.59 | 0.0001 |  | 0.72 | 0.20-2.54 | 0.6104 |
| ***Yorkshire & Humber*** | 0.19 | 0.02-1.69 | 0.1372 |  | 0.36 | 0.17-0.79 | 0.0112 |  | 0.19 | 0.02-1.89 | 0.1566 |
| ***East Midlands*** | 0.26 | 0.03-2.25 | 0.2237 |  | 0.80 | 0.39-1.62 | 0.5329 |  | 0.32 | 0.03-3.21 | 0.3351 |
| ***West Midlands*** | 0.44 | 0.15-1.27 | 0.1301 |  | 0.45 | 0.26-0.76 | 0.0030 |  | 0.71 | 0.19-2.64 | 0.6111 |
| ***East of England*** | 0.42 | 0.14-1.27 | 0.1233 |  | 0.46 | 0.26-0.80 | 0.0059 |  | 0.70 | 0.18-2.64 | 0.5943 |
| ***South West*** | 0.24 | 0.08-0.74 | 0.0130 |  | 0.45 | 0.27-0.76 | 0.0027 |  | 0.32 | 0.08-1.32 | 0.1158 |
| ***South Central*** | 0.50 | 0.17-1.50 | 0.2163 |  | 0.69 | 0.41-1.15 | 0.1508 |  | 0.51 | 0.13-2.02 | 0.3356 |
| ***London*** | 0.37 | 0.13-1.03 | 0.0572 |  | 0.48 | 0.29-0.81 | 0.0058 |  | 0.79 | 0.23-2.78 | 0.7156 |
| ***South East Coast*** | 0.36 | 0.12-1.04 | 0.0594 |  | 0.35 | 0.20-0.62 | 0.0002 |  | 0.88 | 0.25-3.17 | 0.8499 |
| ***BMI: Underweight*** | N/A | N/A | N/A |  | 0.84 | 0.26-2.72 | 0.7763 |  | N/A | N/A | N/A |
| ***BMI: Overweight*** | N/A | N/A | N/A |  | 0.63 | 0.20-2.02 | 0.4394 |  | N/A | N/A | N/A |
| ***BMI: Obese*** | N/A | N/A | N/A |  | 0.53 | 0.17-1.69 | 0.2830 |  | N/A | N/A | N/A |
| ***BMI: Missing*** | N/A | N/A | N/A |  | 0.91 | 0.22-3.68 | 0.8922 |  | N/A | N/A | N/A |
| ***Ex-Smoker*** | 1.18 | 0.68-2.03 | 0.5561 |  | 0.83 | 0.65-1.07 | 0.1568 |  | 0.90 | 0.54-1.51 | 0.6998 |
| ***Current Smoker*** | 0.99 | 0.49-1.99 | 0.9844 |  | 0.64 | 0.44-0.93 | 0.0206 |  | 0.95 | 0.50-1.80 | 0.8696 |
| ***Smoking: Missing*** | 3.43 | 0.43-27.32 | 0.2443 |  | 1.56 | 0.57-4.28 | 0.3890 |  | 5.90 | 0.74-46.76 | 0.0929 |
| ***Alcohol: 1-14*** | 0.64 | 0.34-1.21 | 0.1700 |  | 0.80 | 0.60-1.07 | 0.1265 |  | 0.90 | 0.49-1.62 | 0.7185 |
| ***Alcohol: 15-42*** | 0.91 | 0.38-2.20 | 0.8391 |  | 0.71 | 0.43-1.17 | 0.1787 |  | 1.06 | 0.44-2.57 | 0.8974 |
| ***Alcohol: >42*** | 0.59 | 0.13-2.77 | 0.5057 |  | 0.32 | 0.08-1.31 | 0.1139 |  | 0.40 | 0.05-3.18 | 0.3869 |
| ***Alcohol: Missing*** | 0.38 | 0.15-0.96 | 0.0399 |  | 0.83 | 0.57-1.21 | 0.3265 |  | 0.43 | 0.19-0.95 | 0.0373 |
| ***Morbidities*** | 0.71 | 0.58-0.86 | 0.0006 |  | 0.86 | 0.79-0.94 | 0.0006 |  | 0.86 | 0.72-1.02 | 0.0862 |
| ***Prescriptions*** | 0.98 | 0.94-1.02 | 0.3007 |  | 0.99 | 0.98-1.01 | 0.4440 |  | 1.00 | 0.97-1.03 | 0.9223 |
| ***Hospitalisations*** | 1.23 | 0.88-1.70 | 0.2216 |  | 1.14 | 0.97-1.35 | 0.1165 |  | 1.13 | 0.81-1.57 | 0.4739 |
| ***Duration of diabetes (years)*** | 1.01 | 0.97-1.05 | 0.6224 |  | 0.99 | 0.97-1.01 | 0.3351 |  | 1.01 | 0.97-1.05 | 0.5859 |
| ***Duration of retinopathy (years)*** | 0.98 | 0.92-1.04 | 0.5462 |  | 0.99 | 0.96-1.02 | 0.5917 |  | 1.00 | 0.95-1.05 | 0.8891 |
| ***Complications*** | 1.70 | 1.33-2.18 | <0.0001 |  | 1.31 | 1.18-1.46 | <0.0001 |  | 1.39 | 1.10-1.76 | 0.0054 |
| ***Glucose lowering therapies*** | 1.60 | 1.19-2.14 | 0.0018 |  | 1.38 | 1.22-1.56 | <0.0001 |  | 1.06 | 0.81-1.40 | 0.6713 |
| ***Insulin prescription*** | 1.26 | 0.71-2.22 | 0.4264 |  | 1.67 | 1.30-2.15 | 0.0001 |  | 2.32 | 1.37-3.93 | 0.0017 |
|  |  |  |  |  |  |  |  |  |  |  |  |
|  | **Value** | **95% CI (lower)** | **95% CI (upper)** |  | **Value** | **95% CI (lower)** | **95% CI (upper)** |  | **Value** | **95% CI (lower)** | **95% CI (upper)** |
| ***n after matching*** | 1,396 |  |  |  | 5,342 |  |  |  | 1,406 |  |  |
| ***C-statistic*** | 0.7388 | 0.7375 | 0.7402 |  | 0.6997 | 0.6993 | 0.7001 |  | 0.7135 | 0.7120 | 0.7150 |

Reference groups for categorical covariates include: white (ethnicity); North East (region); normal weight (BMI); non-smoker (smoking status); 0 units (alcohol consumption); and no insulin prescription (insulin use). N/A indicates that the sample after propensity score matching did not contain observations for the covariate.

Table S7: Multivariate hazard ratios (corresponding 95% CIs and p-values) for risk of sight-threatening diabetic retinopathy by each covariate across QOF and NDA exposure definitions after 1:1 propensity score matching, including the adjusted study size (n), and C-statistic (also with corresponding 95% CI).

|  | **Exposure Definition** | | | | | | | | | | |
| --- | --- | --- | --- | --- | --- | --- | --- | --- | --- | --- | --- |
|  | **Achieve All QOF Targets** | | |  | **Meet All NDA Processes** | | |  | **Achieve All QOF & NDA Targets** | | |
|  | **Hazard Ratio** | **95% CI** | **p** |  | **Hazard Ratio** | **95% CI** | **p** |  | **Hazard Ratio** | **95% CI** | **p** |
| ***Exposure*** | 0.85 | 0.72-1.01 | 0.0659 |  | 0.87 | 0.76-1.01 | 0.0594 |  | 0.81 | 0.66-0.98 | 0.0280 |
| ***Age*** | 1.00 | 0.99-1.01 | 0.4531 |  | 0.99 | 0.98-0.99 | 0.0002 |  | 0.99 | 0.98-1.00 | 0.1807 |
| ***Sex: Female*** | 1.01 | 0.84-1.23 | 0.8919 |  | 1.17 | 1.00-1.37 | 0.0550 |  | 1.15 | 0.92-1.44 | 0.2061 |
| ***Ethnicity: Asian*** | 1.42 | 1.05-1.93 | 0.0225 |  | 1.12 | 0.87-1.45 | 0.3692 |  | 1.45 | 1.02-2.07 | 0.0379 |
| ***Ethnicity: Black*** | 1.14 | 0.62-2.08 | 0.6727 |  | 1.33 | 0.91-1.95 | 0.1345 |  | 1.28 | 0.65-2.53 | 0.4733 |
| ***Ethnicity: Mixed*** | 0.99 | 0.41-2.40 | 0.9812 |  | 1.19 | 0.56-2.51 | 0.6541 |  | 0.63 | 0.16-2.55 | 0.5195 |
| ***Ethnicity: Other*** | 0.89 | 0.42-1.90 | 0.7637 |  | 1.03 | 0.60-1.75 | 0.9281 |  | 1.30 | 0.61-2.78 | 0.5034 |
| ***IMD*** | 1.00 | 0.99-1.02 | 0.6167 |  | 1.00 | 0.99-1.01 | 0.8710 |  | 1.00 | 0.99-1.02 | 0.6582 |
| ***North West*** | 0.71 | 0.42-1.20 | 0.1964 |  | 0.63 | 0.43-0.92 | 0.0176 |  | 0.79 | 0.40-1.55 | 0.4918 |
| ***Yorkshire & Humber*** | 1.08 | 0.59-1.97 | 0.8077 |  | 0.79 | 0.49-1.29 | 0.3477 |  | 1.30 | 0.62-2.73 | 0.4955 |
| ***East Midlands*** | 1.26 | 0.65-2.46 | 0.4929 |  | 0.84 | 0.49-1.45 | 0.5262 |  | 1.98 | 0.90-4.35 | 0.0875 |
| ***West Midlands*** | 0.81 | 0.47-1.39 | 0.4478 |  | 0.65 | 0.43-0.96 | 0.0308 |  | 1.13 | 0.57-2.24 | 0.7215 |
| ***East of England*** | 0.88 | 0.51-1.51 | 0.6344 |  | 0.69 | 0.46-1.04 | 0.0729 |  | 1.01 | 0.50-2.02 | 0.9821 |
| ***South West*** | 1.03 | 0.61-1.74 | 0.9009 |  | 0.73 | 0.50-1.08 | 0.1122 |  | 1.32 | 0.68-2.57 | 0.4087 |
| ***South Central*** | 0.77 | 0.44-1.33 | 0.3443 |  | 0.69 | 0.47-1.03 | 0.0732 |  | 0.90 | 0.45-1.80 | 0.7630 |
| ***London*** | 0.78 | 0.46-1.32 | 0.3510 |  | 0.66 | 0.45-0.97 | 0.0364 |  | 1.02 | 0.52-1.99 | 0.9629 |
| ***South East Coast*** | 0.83 | 0.49-1.43 | 0.5096 |  | 0.63 | 0.42-0.93 | 0.0214 |  | 1.02 | 0.52-2.03 | 0.9484 |
| ***BMI: Underweight*** | 1.36 | 0.43-4.30 | 0.6010 |  | 1.59 | 0.50-5.03 | 0.4270 |  | 1.84 | 0.45-7.50 | 0.3975 |
| ***BMI: Overweight*** | 1.43 | 0.46-4.47 | 0.5418 |  | 1.83 | 0.58-5.71 | 0.3000 |  | 1.91 | 0.47-7.72 | 0.3642 |
| ***BMI: Obese*** | 1.31 | 0.42-4.12 | 0.6408 |  | 1.58 | 0.51-4.95 | 0.4288 |  | 1.71 | 0.42-6.90 | 0.4544 |
| ***BMI: Missing*** | 2.68 | 0.71-10.15 | 0.1475 |  | 2.33 | 0.64-8.51 | 0.1998 |  | 0.66 | 0.06-7.36 | 0.7387 |
| ***Ex-Smoker*** | 0.83 | 0.69-1.00 | 0.0464 |  | 0.82 | 0.70-0.96 | 0.0153 |  | 0.85 | 0.68-1.06 | 0.1406 |
| ***Current Smoker*** | 0.63 | 0.46-0.86 | 0.0037 |  | 0.74 | 0.58-0.93 | 0.0114 |  | 0.84 | 0.61-1.16 | 0.2952 |
| ***Smoking: Missing*** | 0.95 | 0.30-2.99 | 0.9331 |  | 7.59 | 1.05-55.12 | 0.0450 |  | 4.23 | 0.56-31.84 | 0.1609 |
| ***Alcohol: 1-14*** | 0.92 | 0.73-1.16 | 0.4698 |  | 0.83 | 0.69-1.00 | 0.0528 |  | 0.79 | 0.61-1.03 | 0.0770 |
| ***Alcohol: 15-42*** | 1.02 | 0.72-1.44 | 0.9263 |  | 0.89 | 0.67-1.20 | 0.4495 |  | 1.11 | 0.76-1.62 | 0.5832 |
| ***Alcohol: >42*** | 1.26 | 0.65-2.45 | 0.4916 |  | 0.45 | 0.21-0.96 | 0.0396 |  | 1.27 | 0.61-2.65 | 0.5309 |
| ***Alcohol: Missing*** | 1.25 | 0.93-1.68 | 0.1473 |  | 0.91 | 0.72-1.16 | 0.4535 |  | 1.12 | 0.79-1.58 | 0.5383 |
| ***Morbidities*** | 0.92 | 0.87-0.99 | 0.0168 |  | 0.90 | 0.85-0.95 | 0.0003 |  | 0.92 | 0.85-0.99 | 0.0219 |
| ***Prescriptions*** | 1.00 | 0.99-1.01 | 0.7423 |  | 0.99 | 0.98-1.00 | 0.0267 |  | 1.01 | 1.00-1.02 | 0.2048 |
| ***Hospitalisations*** | 1.16 | 1.01-1.33 | 0.0324 |  | 1.16 | 1.04-1.30 | 0.0067 |  | 1.12 | 0.93-1.36 | 0.2224 |
| ***Duration of diabetes (years)*** | 1.01 | 1.00-1.03 | 0.0405 |  | 1.01 | 1.00-1.02 | 0.0777 |  | 1.02 | 1.00-1.03 | 0.0105 |
| ***Duration of retinopathy (years)*** | 1.00 | 0.98-1.03 | 0.7435 |  | 1.00 | 0.98-1.02 | 0.9328 |  | 0.99 | 0.96-1.02 | 0.4444 |
| ***Complications*** | 1.14 | 1.04-1.24 | 0.0044 |  | 1.24 | 1.16-1.34 | <0.0001 |  | 1.22 | 1.10-1.35 | 0.0001 |
| ***Glucose lowering therapies*** | 1.28 | 1.16-1.43 | <0.0001 |  | 1.31 | 1.21-1.42 | <0.0001 |  | 1.29 | 1.15-1.45 | <0.0001 |
| ***Insulin prescription*** | 1.81 | 1.45-2.25 | <0.0001 |  | 1.77 | 1.51-2.09 | <0.0001 |  | 1.93 | 1.51-2.47 | <0.0001 |
|  |  |  |  |  |  |  |  |  |  |  |  |
|  | **Value** | **95% CI (lower)** | **95% CI (upper)** |  | **Value** | **95% CI (lower)** | **95% CI (upper)** |  | **Value** | **95% CI (lower)** | **95% CI (upper)** |
| ***n after matching*** | 12,608 |  |  |  | 13,996 |  |  |  | 9,228 |  |  |
| ***C-statistic*** | 0.6577 | 0.6574 | 0.6580 |  | 0.6773 | 0.6771 | 0.6775 |  | 0.6896 | 0.6893 | 0.6900 |

Reference groups for categorical covariates include: white (ethnicity); North East (region); normal weight (BMI); non-smoker (smoking status); 0 units (alcohol consumption); and no insulin prescription (insulin use).

Table S8: Univariate hazard ratios (with corresponding 95% CIs and p-values) for risk of sight-threatening diabetic retinopathy by each covariate across QOF exposure definitions, *among those who meet all other QOF targets*, after 1:1 propensity score matching.

|  | **Exposure Definition** | | | | | | | | | | |
| --- | --- | --- | --- | --- | --- | --- | --- | --- | --- | --- | --- |
|  | **Achieve HBA1c QOF**  **Target** | | |  | **Achieve Blood Pressure QOF Target** | | |  | **Achieve Cholesterol QOF**  **Target** | | |
|  | **Hazard Ratio** | **95% CI** | **p** |  | **Hazard Ratio** | **95% CI** | **p** |  | **Hazard Ratio** | **95% CI** | **p** |
| ***Exposure*** | 0.78 | 0.62-0.97 | 0.0255 |  | 1.10 | 0.87-1.38 | 0.4264 |  | 1.35 | 0.86-2.11 | 0.1894 |
| ***Age*** | 1.00 | 0.99-1.00 | 0.3440 |  | 1.00 | 0.99-1.01 | 0.7243 |  | 1.01 | 0.99-1.03 | 0.4465 |
| ***Sex: Female*** | 1.04 | 0.83-1.30 | 0.7130 |  | 1.11 | 0.88-1.40 | 0.3829 |  | 1.04 | 0.66-1.61 | 0.8778 |
| ***Ethnicity: Asian*** | 1.80 | 1.30-2.48 | 0.0004 |  | 1.59 | 1.07-2.36 | 0.0227 |  | 1.57 | 0.68-3.61 | 0.2882 |
| ***Ethnicity: Black*** | 0.51 | 0.16-1.58 | 0.2409 |  | 2.14 | 1.17-3.90 | 0.0136 |  | 2.29 | 0.99-5.26 | 0.0517 |
| ***Ethnicity: Mixed*** | N/A | N/A | N/A |  | 1.04 | 0.26-4.17 | 0.9586 |  | 1.63 | 0.23-11.71 | 0.6277 |
| ***Ethnicity: Other*** | 0.74 | 0.24-2.30 | 0.6000 |  | 1.00 | 0.37-2.68 | 0.9991 |  | N/A | N/A | N/A |
| ***IMD*** | 1.00 | 0.98-1.02 | 0.6929 |  | 1.00 | 0.98-1.02 | 0.8948 |  | 0.99 | 0.95-1.03 | 0.6261 |
| ***North West*** | 0.90 | 0.66-1.22 | 0.4978 |  | 0.80 | 0.58-1.11 | 0.1819 |  | 0.67 | 0.34-1.30 | 0.2323 |
| ***Yorkshire & Humber*** | 1.25 | 0.75-2.10 | 0.3943 |  | 1.86 | 1.20-2.91 | 0.0059 |  | 0.95 | 0.30-3.01 | 0.9296 |
| ***East Midlands*** | 0.86 | 0.38-1.92 | 0.7066 |  | 1.17 | 0.58-2.36 | 0.6644 |  | 0.59 | 0.08-4.27 | 0.6042 |
| ***West Midlands*** | 0.76 | 0.52-1.11 | 0.1597 |  | 0.97 | 0.67-1.40 | 0.8736 |  | 0.64 | 0.28-1.46 | 0.2859 |
| ***East of England*** | 0.64 | 0.41-0.99 | 0.0430 |  | 1.19 | 0.84-1.69 | 0.3356 |  | 1.19 | 0.60-2.39 | 0.6152 |
| ***South West*** | 1.70 | 1.30-2.24 | 0.0001 |  | 0.85 | 0.60-1.20 | 0.3518 |  | 1.61 | 0.91-2.82 | 0.0990 |
| ***South Central*** | 0.88 | 0.62-1.26 | 0.4886 |  | 1.06 | 0.75-1.52 | 0.7273 |  | 1.18 | 0.61-2.28 | 0.6324 |
| ***London*** | 1.03 | 0.75-1.41 | 0.8634 |  | 0.86 | 0.60-1.22 | 0.4027 |  | 1.08 | 0.58-1.99 | 0.8171 |
| ***South East Coast*** | 0.89 | 0.63-1.26 | 0.5094 |  | 1.12 | 0.80-1.56 | 0.5187 |  | 1.01 | 0.53-1.91 | 0.9745 |
| ***BMI: Underweight*** | 0.89 | 0.64-1.24 | 0.5014 |  | 1.22 | 0.91-1.65 | 0.1902 |  | 1.04 | 0.60-1.80 | 0.8879 |
| ***BMI: Overweight*** | 1.20 | 0.96-1.51 | 0.1115 |  | 0.96 | 0.75-1.22 | 0.7362 |  | 1.01 | 0.64-1.60 | 0.9670 |
| ***BMI: Obese*** | 0.91 | 0.73-1.13 | 0.3984 |  | 0.91 | 0.73-1.15 | 0.4416 |  | 0.99 | 0.63-1.54 | 0.9609 |
| ***BMI: Missing*** | 0.71 | 0.18-2.85 | 0.6303 |  | 2.06 | 0.85-4.98 | 0.1096 |  | 2.04 | 0.28-14.66 | 0.4795 |
| ***Ex-Smoker*** | 0.88 | 0.70-1.11 | 0.2797 |  | 0.81 | 0.64-1.04 | 0.0947 |  | 0.88 | 0.55-1.39 | 0.5841 |
| ***Current Smoker*** | 0.92 | 0.65-1.30 | 0.6299 |  | 0.74 | 0.50-1.11 | 0.1437 |  | 0.43 | 0.17-1.05 | 0.0644 |
| ***Smoking: Missing*** | 0.91 | 0.23-3.65 | 0.8915 |  | N/A | N/A | N/A |  | 1.28 | 0.18-9.20 | 0.8092 |
| ***Alcohol: 1-14*** | 0.82 | 0.66-1.02 | 0.0772 |  | 0.90 | 0.71-1.14 | 0.3777 |  | 0.51 | 0.33-0.80 | 0.0031 |
| ***Alcohol: 15-42*** | 0.82 | 0.54-1.26 | 0.3739 |  | 0.69 | 0.44-1.06 | 0.0886 |  | 1.47 | 0.78-2.78 | 0.2361 |
| ***Alcohol: >42*** | 0.75 | 0.28-2.01 | 0.5699 |  | 0.84 | 0.35-2.03 | 0.6979 |  | 0.67 | 0.09-4.79 | 0.6860 |
| ***Alcohol: Missing*** | 1.19 | 0.87-1.62 | 0.2802 |  | 1.32 | 0.95-1.85 | 0.1010 |  | 2.04 | 1.18-3.54 | 0.0107 |
| ***Morbidities*** | 1.03 | 0.97-1.10 | 0.3669 |  | 1.00 | 0.93-1.08 | 0.9620 |  | 1.03 | 0.90-1.17 | 0.7079 |
| ***Prescriptions*** | 1.01 | 1.00-1.02 | 0.1302 |  | 1.02 | 1.01-1.03 | 0.0019 |  | 1.01 | 0.99-1.04 | 0.2081 |
| ***Hospitalisations*** | 1.18 | 1.00-1.39 | 0.0534 |  | 1.19 | 0.99-1.43 | 0.0621 |  | 1.18 | 0.88-1.59 | 0.2710 |
| ***Duration of diabetes (years)*** | 1.01 | 1.00-1.03 | 0.1536 |  | 1.03 | 1.01-1.04 | 0.0042 |  | 1.00 | 0.96-1.04 | 0.8884 |
| ***Duration of retinopathy (years)*** | 1.00 | 0.97-1.03 | 0.9241 |  | 0.99 | 0.96-1.02 | 0.6228 |  | 1.00 | 0.94-1.06 | 0.9441 |
| ***Complications*** | 1.13 | 1.03-1.23 | 0.0088 |  | 1.18 | 1.07-1.29 | 0.0007 |  | 1.25 | 1.05-1.50 | 0.0130 |
| ***Glucose lowering therapies*** | 1.14 | 1.02-1.29 | 0.0275 |  | 1.35 | 1.21-1.52 | <0.0001 |  | 1.42 | 1.16-1.74 | 0.0008 |
| ***Insulin prescription*** | 1.96 | 1.57-2.45 | <0.0001 |  | 2.20 | 1.67-2.89 | <0.0001 |  | 1.53 | 0.85-2.78 | 0.1595 |

Study sizes across exposures after 1:1 propensity score matching are found in Table S9, as they are the same between univariate and multivariate analyses.N/A indicates that the sample after propensity score matching did not contain observations for the covariate.

Table S9: Multivariate hazard ratios (with corresponding 95% CIs and p-values) for risk of sight-threatening diabetic retinopathy by each covariate across QOF exposure definitions, *among those who meet all other QOF targets*, after 1:1 propensity score matching, including the adjusted study size (n) and C-statistic (also with corresponding 95% CI).

|  | **Exposure Definition** | | | | | | | | | | |
| --- | --- | --- | --- | --- | --- | --- | --- | --- | --- | --- | --- |
|  | **Achieve HbA1c QOF Target** | | |  | **Achieve Blood Pressure QOF Target** | | |  | **Achieve Cholesterol QOF Target** | | |
|  | **Hazard Ratio** | **95% CI** | **p** |  | **Hazard Ratio** | **95% CI** | **p** |  | **Hazard Ratio** | **95% CI** | **p** |
| ***Exposure*** | 0.83 | 0.66-1.04 | 0.0980 |  | 1.08 | 0.85-1.36 | 0.5360 |  | 1.34 | 0.85-2.11 | 0.2061 |
| ***Age*** | 0.99 | 0.98-1.01 | 0.2937 |  | 1.00 | 0.99-1.01 | 0.9372 |  | 1.00 | 0.98-1.03 | 0.7869 |
| ***Sex: Female*** | 1.06 | 0.82-1.36 | 0.6763 |  | 1.20 | 0.92-1.57 | 0.1799 |  | 1.25 | 0.73-2.17 | 0.4178 |
| ***Ethnicity: Asian*** | 1.49 | 1.04-2.14 | 0.0316 |  | 1.26 | 0.82-1.95 | 0.2930 |  | 1.03 | 0.41-2.59 | 0.9581 |
| ***Ethnicity: Black*** | 0.52 | 0.17-1.63 | 0.2630 |  | 1.90 | 1.02-3.52 | 0.0419 |  | 2.09 | 0.87-5.05 | 0.1013 |
| ***Ethnicity: Mixed*** | N/A | N/A | N/A |  | 0.94 | 0.23-3.80 | 0.9289 |  | 2.40 | 0.32-17.82 | 0.3931 |
| ***Ethnicity: Other*** | 0.71 | 0.22-2.23 | 0.5536 |  | 0.97 | 0.36-2.66 | 0.9600 |  | N/A | N/A | N/A |
| ***IMD*** | 0.99 | 0.97-1.01 | 0.5270 |  | 1.00 | 0.98-1.03 | 0.6783 |  | 0.99 | 0.95-1.04 | 0.7231 |
| ***North West*** | 0.53 | 0.29-0.97 | 0.0381 |  | 0.95 | 0.43-2.12 | 0.9007 |  | 0.85 | 0.18-3.92 | 0.8351 |
| ***Yorkshire & Humber*** | 0.69 | 0.33-1.43 | 0.3148 |  | 2.11 | 0.89-4.96 | 0.0890 |  | 1.16 | 0.19-7.11 | 0.8720 |
| ***East Midlands*** | 0.50 | 0.19-1.30 | 0.1559 |  | 1.33 | 0.48-3.68 | 0.5856 |  | 0.70 | 0.06-7.87 | 0.7751 |
| ***West Midlands*** | 0.45 | 0.24-0.86 | 0.0157 |  | 1.11 | 0.49-2.53 | 0.7998 |  | 0.79 | 0.16-3.97 | 0.7740 |
| ***East of England*** | 0.37 | 0.19-0.73 | 0.0043 |  | 1.33 | 0.59-3.01 | 0.4946 |  | 1.29 | 0.27-6.09 | 0.7478 |
| ***South West*** | 0.89 | 0.49-1.59 | 0.6835 |  | 0.99 | 0.44-2.23 | 0.9776 |  | 1.62 | 0.36-7.24 | 0.5276 |
| ***South Central*** | 0.49 | 0.26-0.92 | 0.0268 |  | 1.19 | 0.53-2.70 | 0.6749 |  | 1.26 | 0.27-5.97 | 0.7669 |
| ***London*** | 0.58 | 0.31-1.06 | 0.0741 |  | 0.97 | 0.43-2.19 | 0.9445 |  | 1.24 | 0.27-5.61 | 0.7829 |
| ***South East Coast*** | 0.49 | 0.26-0.91 | 0.0241 |  | 1.24 | 0.55-2.78 | 0.6068 |  | 1.13 | 0.24-5.27 | 0.8724 |
| ***BMI: Underweight*** | 1.16 | 0.28-4.84 | 0.8356 |  | 2.21 | 0.30-16.04 | 0.4335 |  | N/A | N/A | N/A |
| ***BMI: Overweight*** | 1.48 | 0.36-6.02 | 0.5849 |  | 1.86 | 0.26-13.37 | 0.5385 |  | N/A | N/A | N/A |
| ***BMI: Obese*** | 1.17 | 0.29-4.76 | 0.8264 |  | 1.74 | 0.24-12.51 | 0.5819 |  | N/A | N/A | N/A |
| ***BMI: Missing*** | 1.01 | 0.14-7.22 | 0.9938 |  | 3.61 | 0.42-31.17 | 0.2428 |  | N/A | N/A | N/A |
| ***Ex-Smoker*** | 0.88 | 0.68-1.13 | 0.3167 |  | 0.81 | 0.63-1.06 | 0.1267 |  | 0.80 | 0.48-1.34 | 0.4006 |
| ***Current Smoker*** | 0.83 | 0.57-1.21 | 0.3420 |  | 0.71 | 0.47-1.08 | 0.1138 |  | 0.38 | 0.15-0.97 | 0.0438 |
| ***Smoking: Missing*** | 0.92 | 0.22-3.76 | 0.9069 |  | N/A | N/A | N/A |  | 1.41 | 0.19-10.75 | 0.7401 |
| ***Alcohol: 1-14*** | 0.79 | 0.59-1.05 | 0.1064 |  | 0.87 | 0.64-1.19 | 0.3767 |  | 0.58 | 0.31-1.08 | 0.0833 |
| ***Alcohol: 15-42*** | 0.71 | 0.43-1.16 | 0.1732 |  | 0.72 | 0.43-1.20 | 0.2072 |  | 1.48 | 0.63-3.50 | 0.3722 |
| ***Alcohol: >42*** | 0.67 | 0.24-1.87 | 0.4499 |  | 0.98 | 0.39-2.49 | 0.9658 |  | 0.59 | 0.07-4.71 | 0.6201 |
| ***Alcohol: Missing*** | 0.91 | 0.62-1.33 | 0.6235 |  | 1.04 | 0.69-1.57 | 0.8472 |  | 1.47 | 0.72-3.01 | 0.2949 |
| ***Morbidities*** | 0.98 | 0.90-1.07 | 0.6504 |  | 0.91 | 0.83-1.00 | 0.0551 |  | 0.93 | 0.78-1.10 | 0.3745 |
| ***Prescriptions*** | 0.99 | 0.98-1.01 | 0.5310 |  | 1.00 | 0.98-1.01 | 0.7963 |  | 0.98 | 0.94-1.03 | 0.4080 |
| ***Hospitalisations*** | 1.14 | 0.96-1.37 | 0.1437 |  | 1.13 | 0.93-1.37 | 0.2224 |  | 1.14 | 0.84-1.56 | 0.3936 |
| ***Duration of diabetes (years)*** | 1.00 | 0.99-1.02 | 0.6134 |  | 1.02 | 1.00-1.04 | 0.0776 |  | 0.99 | 0.95-1.03 | 0.4897 |
| ***Duration of retinopathy (years)*** | 1.00 | 0.97-1.03 | 0.9815 |  | 0.99 | 0.95-1.02 | 0.4157 |  | 0.99 | 0.93-1.06 | 0.8009 |
| ***Complications*** | 1.12 | 1.00-1.26 | 0.0570 |  | 1.24 | 1.10-1.40 | 0.0005 |  | 1.32 | 1.06-1.66 | 0.0152 |
| ***Glucose lowering therapies*** | 1.13 | 0.98-1.30 | 0.0874 |  | 1.26 | 1.09-1.45 | 0.0021 |  | 1.59 | 1.19-2.12 | 0.0018 |
| ***Insulin prescription*** | 1.79 | 1.40-2.27 | <0.0001 |  | 1.60 | 1.18-2.18 | 0.0026 |  | 1.12 | 0.57-2.19 | 0.7353 |
|  |  |  |  |  |  |  |  |  |  |  |  |
|  | **Value** | **95% CI (lower)** | **95% CI (upper)** |  | **Value** | **95% CI (lower)** | **95% CI (upper)** |  | **Value** | **95% CI (lower)** | **95% CI (upper)** |
| ***n after matching*** | 5,306 |  |  |  | 7,208 |  |  |  | 2,308 |  |  |
| ***C-statistic*** | 0.6609 | 0.6605 | 0.6614 |  | 0.6727 | 0.6723 | 0.6732 |  | 0.7026 | 0.7008 | 0.7044 |

Reference groups for categorical covariates include: white (ethnicity); North East (region); normal weight (BMI); non-smoker (smoking status); 0 units (alcohol consumption); and no insulin prescription (insulin use). N/A indicates that the sample after propensity score matching did not contain observations for the covariate.

Table S10: Univariate hazard ratios (with corresponding 95% CIs and p-values) for risk of sight-threatening diabetic retinopathy by each covariate across QOF exposure definitions, *among those who meet none of the other QOF targets*, after 1:1 propensity score matching.

|  | **Exposure Definition** | | | | | | | | | | |
| --- | --- | --- | --- | --- | --- | --- | --- | --- | --- | --- | --- |
|  | **Achieve HBA1c QOF**  **Target** | | |  | **Achieve Blood Pressure QOF Target** | | |  | **Achieve Cholesterol QOF**  **Target** | | |
|  | **Hazard Ratio** | **95% CI** | **p** |  | **Hazard Ratio** | **95% CI** | **p** |  | **Hazard Ratio** | **95% CI** | **p** |
| ***Exposure*** | 0.43 | 0.28-0.65 | 0.0001 |  | 0.61 | 0.44-0.83 | 0.0020 |  | 0.79 | 0.60-1.04 | 0.0872 |
| ***Age*** | 0.99 | 0.98-1.01 | 0.2124 |  | 0.99 | 0.98-1.00 | 0.0382 |  | 0.98 | 0.97-1.00 | 0.0051 |
| ***Sex: Female*** | 1.14 | 0.77-1.69 | 0.5048 |  | 0.89 | 0.66-1.21 | 0.4616 |  | 1.08 | 0.82-1.42 | 0.5680 |
| ***Ethnicity: Asian*** | 1.35 | 0.63-2.91 | 0.4427 |  | 1.44 | 0.84-2.44 | 0.1817 |  | 1.52 | 0.98-2.37 | 0.0625 |
| ***Ethnicity: Black*** | 1.69 | 0.69-4.14 | 0.2552 |  | 0.52 | 0.13-2.08 | 0.3521 |  | 0.80 | 0.36-1.81 | 0.5967 |
| ***Ethnicity: Mixed*** | 2.84 | 0.70-11.52 | 0.1446 |  | 6.86 | 2.80-16.76 | <0.0001 |  | 1.59 | 0.51-4.97 | 0.4251 |
| ***Ethnicity: Other*** | 1.36 | 0.34-5.51 | 0.6680 |  | 1.84 | 0.76-4.48 | 0.1795 |  | 1.58 | 0.59-4.24 | 0.3678 |
| ***IMD*** | 1.03 | 0.99-1.06 | 0.1506 |  | 1.01 | 0.98-1.04 | 0.3835 |  | 0.99 | 0.96-1.01 | 0.2416 |
| ***North West*** | 1.10 | 0.67-1.80 | 0.6999 |  | 0.88 | 0.58-1.33 | 0.5399 |  | 0.92 | 0.64-1.34 | 0.6767 |
| ***Yorkshire & Humber*** | 0.69 | 0.22-2.18 | 0.5298 |  | 0.50 | 0.16-1.57 | 0.2355 |  | 1.08 | 0.51-2.30 | 0.8336 |
| ***East Midlands*** | 1.62 | 0.51-5.10 | 0.4127 |  | 2.23 | 0.92-5.44 | 0.0768 |  | 0.84 | 0.31-2.26 | 0.7328 |
| ***West Midlands*** | 1.43 | 0.85-2.41 | 0.1774 |  | 0.78 | 0.48-1.28 | 0.3240 |  | 0.78 | 0.50-1.22 | 0.2737 |
| ***East of England*** | 1.12 | 0.60-2.09 | 0.7287 |  | 0.90 | 0.54-1.51 | 0.7027 |  | 1.10 | 0.71-1.69 | 0.6702 |
| ***South West*** | 0.47 | 0.22-1.02 | 0.0565 |  | 0.97 | 0.62-1.52 | 0.8853 |  | 0.92 | 0.61-1.37 | 0.6752 |
| ***South Central*** | 0.95 | 0.51-1.77 | 0.8685 |  | 1.31 | 0.83-2.05 | 0.2437 |  | 1.42 | 0.98-2.05 | 0.0624 |
| ***London*** | 0.65 | 0.33-1.30 | 0.2244 |  | 1.00 | 0.65-1.56 | 0.9864 |  | 0.69 | 0.43-1.09 | 0.1128 |
| ***South East Coast*** | 0.97 | 0.53-1.77 | 0.9121 |  | 0.88 | 0.54-1.44 | 0.6203 |  | 1.02 | 0.67-1.53 | 0.9328 |
| ***BMI: Underweight*** | 0.80 | 0.42-1.54 | 0.5110 |  | 0.68 | 0.40-1.13 | 0.1391 |  | 0.63 | 0.37-1.09 | 0.0959 |
| ***BMI: Overweight*** | 1.39 | 0.93-2.08 | 0.1063 |  | 1.21 | 0.88-1.66 | 0.2319 |  | 1.28 | 0.96-1.71 | 0.0889 |
| ***BMI: Obese*** | 0.78 | 0.53-1.16 | 0.2238 |  | 0.98 | 0.72-1.33 | 0.8971 |  | 0.90 | 0.69-1.19 | 0.4680 |
| ***BMI: Missing*** | 3.23 | 0.79-13.14 | 0.1015 |  | 1.25 | 0.18-8.94 | 0.8230 |  | 1.68 | 0.54-5.26 | 0.3718 |
| ***Ex-Smoker*** | 0.61 | 0.39-0.96 | 0.0330 |  | 0.86 | 0.62-1.20 | 0.3794 |  | 0.65 | 0.48-0.89 | 0.0065 |
| ***Current Smoker*** | 1.18 | 0.69-2.02 | 0.5340 |  | 1.02 | 0.69-1.50 | 0.9220 |  | 1.03 | 0.70-1.52 | 0.8623 |
| ***Smoking: Missing*** | 14.08 | 1.95-101.56 | 0.0087 |  | N/A | N/A | N/A |  | 1.52 | 0.21-10.81 | 0.6785 |
| ***Alcohol: 1-14*** | 0.64 | 0.43-0.94 | 0.0241 |  | 0.78 | 0.58-1.07 | 0.1204 |  | 0.83 | 0.64-1.09 | 0.1901 |
| ***Alcohol: 15-42*** | 2.05 | 1.26-3.35 | 0.0039 |  | 1.40 | 0.85-2.31 | 0.1901 |  | 1.45 | 0.96-2.19 | 0.0770 |
| ***Alcohol: >42*** | 0.68 | 0.17-2.75 | 0.5872 |  | 0.62 | 0.15-2.49 | 0.4971 |  | N/A | N/A | N/A |
| ***Alcohol: Missing*** | 1.30 | 0.76-2.22 | 0.3352 |  | 1.27 | 0.84-1.92 | 0.2546 |  | 1.15 | 0.80-1.65 | 0.4509 |
| ***Morbidities*** | 0.84 | 0.74-0.96 | 0.0122 |  | 0.93 | 0.85-1.03 | 0.1677 |  | 0.96 | 0.88-1.04 | 0.3191 |
| ***Prescriptions*** | 1.01 | 1.00-1.03 | 0.0621 |  | 1.00 | 0.98-1.01 | 0.7144 |  | 1.01 | 0.99-1.02 | 0.4215 |
| ***Hospitalisations*** | 0.99 | 0.73-1.36 | 0.9707 |  | 1.07 | 0.84-1.36 | 0.5827 |  | 1.11 | 0.90-1.37 | 0.3342 |
| ***Duration of diabetes (years)*** | 1.01 | 0.97-1.04 | 0.7436 |  | 1.01 | 0.98-1.04 | 0.4932 |  | 1.03 | 1.01-1.06 | 0.0026 |
| ***Duration of retinopathy (years)*** | 0.96 | 0.91-1.02 | 0.2215 |  | 0.99 | 0.95-1.03 | 0.6406 |  | 0.99 | 0.95-1.03 | 0.5355 |
| ***Complications*** | 1.04 | 0.87-1.23 | 0.6861 |  | 1.19 | 1.05-1.34 | 0.0054 |  | 1.08 | 0.97-1.21 | 0.1756 |
| ***Glucose lowering therapies*** | 1.48 | 1.22-1.81 | 0.0001 |  | 1.09 | 0.94-1.28 | 0.2567 |  | 1.20 | 1.05-1.37 | 0.0082 |
| ***Insulin prescription*** | 2.55 | 1.62-4.01 | 0.0001 |  | 1.82 | 1.34-2.49 | 0.0001 |  | 1.91 | 1.45-2.50 | <0.0001 |

Study sizes across exposures after 1:1 propensity score matching are found in Supplementary Table 10, as they are the same between univariate and multivariate analyses.

N/A indicates that the sample after propensity score matching did not contain observations for the covariate.

Table S11: Multivariate hazard ratios (with corresponding 95% CIs and p-values) for risk of sight-threatening diabetic retinopathy by each covariate across QOF exposure definitions, *among those who meet none of the other QOF targets*, after 1:1 propensity score matching, including the adjusted study size (n) and C-statistic (also with corresponding 95% CI).

|  | **Exposure Definition** | | | | | | | | | | |
| --- | --- | --- | --- | --- | --- | --- | --- | --- | --- | --- | --- |
|  | **Achieve HbA1c QOF Target** | | |  | **Achieve Blood Pressure QOF Target** | | |  | **Achieve Cholesterol QOF Target** | | |
|  | **Hazard Ratio** | **95% CI** | **p** |  | **Hazard Ratio** | **95% CI** | **p** |  | **Hazard Ratio** | **95% CI** | **p** |
| ***Exposure*** | 0.45 | 0.29-0.69 | 0.0003 |  | 0.62 | 0.45-0.85 | 0.0029 |  | 0.74 | 0.56-0.98 | 0.0357 |
| ***Age*** | 0.99 | 0.98-1.01 | 0.5804 |  | 0.99 | 0.97-1.00 | 0.0552 |  | 0.98 | 0.97-1.00 | 0.0112 |
| ***Sex: Female*** | 1.67 | 1.02-2.73 | 0.0430 |  | 1.13 | 0.78-1.62 | 0.5211 |  | 1.23 | 0.89-1.69 | 0.2020 |
| ***Ethnicity: Asian*** | 1.30 | 0.58-2.94 | 0.5226 |  | 1.13 | 0.63-2.03 | 0.6839 |  | 1.22 | 0.75-1.99 | 0.4300 |
| ***Ethnicity: Black*** | 1.13 | 0.43-2.98 | 0.8052 |  | 0.55 | 0.13-2.27 | 0.4067 |  | 0.80 | 0.35-1.83 | 0.5962 |
| ***Ethnicity: Mixed*** | 2.70 | 0.63-11.60 | 0.1821 |  | 7.34 | 2.84-18.95 | <0.0001 |  | 1.65 | 0.52-5.24 | 0.3947 |
| ***Ethnicity: Other*** | 1.00 | 0.23-4.28 | 0.9988 |  | 1.97 | 0.78-4.98 | 0.1538 |  | 1.30 | 0.47-3.61 | 0.6087 |
| ***IMD*** | 1.03 | 0.99-1.07 | 0.1013 |  | 1.01 | 0.98-1.04 | 0.4951 |  | 0.99 | 0.97-1.02 | 0.5966 |
| ***North West*** | 0.53 | 0.23-1.24 | 0.1435 |  | 0.37 | 0.18-0.75 | 0.0065 |  | 0.50 | 0.26-0.95 | 0.0350 |
| ***Yorkshire & Humber*** | 0.28 | 0.07-1.09 | 0.0661 |  | 0.21 | 0.06-0.76 | 0.0175 |  | 0.53 | 0.21-1.34 | 0.1788 |
| ***East Midlands*** | 0.77 | 0.20-2.98 | 0.7075 |  | 0.94 | 0.32-2.76 | 0.9074 |  | 0.36 | 0.12-1.10 | 0.0735 |
| ***West Midlands*** | 0.67 | 0.28-1.62 | 0.3742 |  | 0.32 | 0.15-0.70 | 0.0044 |  | 0.44 | 0.22-0.88 | 0.0209 |
| ***East of England*** | 0.63 | 0.24-1.63 | 0.3412 |  | 0.40 | 0.18-0.89 | 0.0253 |  | 0.59 | 0.30-1.19 | 0.1410 |
| ***South West*** | 0.26 | 0.09-0.74 | 0.0116 |  | 0.36 | 0.17-0.76 | 0.0075 |  | 0.44 | 0.23-0.87 | 0.0176 |
| ***South Central*** | 0.54 | 0.21-1.41 | 0.2100 |  | 0.49 | 0.23-1.04 | 0.0634 |  | 0.66 | 0.34-1.27 | 0.2102 |
| ***London*** | 0.29 | 0.11-0.78 | 0.0141 |  | 0.40 | 0.19-0.84 | 0.0159 |  | 0.37 | 0.18-0.76 | 0.0064 |
| ***South East Coast*** | 0.51 | 0.20-1.31 | 0.1631 |  | 0.35 | 0.16-0.77 | 0.0088 |  | 0.50 | 0.25-0.98 | 0.0448 |
| ***BMI: Underweight*** | N/A | N/A | N/A |  | 0.48 | 0.06-3.76 | 0.4817 |  | 0.44 | 0.06-3.46 | 0.4393 |
| ***BMI: Overweight*** | N/A | N/A | N/A |  | 0.78 | 0.10-5.81 | 0.8042 |  | 0.76 | 0.10-5.59 | 0.7880 |
| ***BMI: Obese*** | N/A | N/A | N/A |  | 0.61 | 0.08-4.60 | 0.6328 |  | 0.57 | 0.08-4.19 | 0.5832 |
| ***BMI: Missing*** | N/A | N/A | N/A |  | 0.97 | 0.06-15.96 | 0.9808 |  | 1.05 | 0.11-10.42 | 0.9661 |
| ***Ex-Smoker*** | 0.72 | 0.44-1.19 | 0.2018 |  | 0.93 | 0.64-1.37 | 0.7249 |  | 0.70 | 0.50-0.98 | 0.0367 |
| ***Current Smoker*** | 1.01 | 0.56-1.82 | 0.9696 |  | 0.91 | 0.59-1.40 | 0.6572 |  | 0.83 | 0.55-1.26 | 0.3907 |
| ***Smoking: Missing*** | 31.27 | 3.67-266.72 | 0.0016 |  | N/A | N/A | N/A |  | 1.51 | 0.21-11.07 | 0.6843 |
| ***Alcohol: 1-14*** | 0.87 | 0.48-1.56 | 0.6314 |  | 0.84 | 0.55-1.28 | 0.4108 |  | 0.95 | 0.65-1.39 | 0.7905 |
| ***Alcohol: 15-42*** | 2.58 | 1.22-5.47 | 0.0134 |  | 1.39 | 0.74-2.62 | 0.3017 |  | 1.54 | 0.90-2.65 | 0.1148 |
| ***Alcohol: >42*** | 1.04 | 0.23-4.70 | 0.9560 |  | 0.47 | 0.11-2.04 | 0.3160 |  | N/A | N/A | N/A |
| ***Alcohol: Missing*** | 1.24 | 0.61-2.54 | 0.5521 |  | 1.13 | 0.66-1.94 | 0.6448 |  | 0.99 | 0.62-1.57 | 0.9585 |
| ***Morbidities*** | 0.79 | 0.66-0.93 | 0.0044 |  | 0.90 | 0.79-1.01 | 0.0803 |  | 0.94 | 0.84-1.05 | 0.2728 |
| ***Prescriptions*** | 1.01 | 0.99-1.03 | 0.4693 |  | 0.99 | 0.96-1.01 | 0.2661 |  | 0.99 | 0.97-1.01 | 0.3219 |
| ***Hospitalisations*** | 1.03 | 0.73-1.46 | 0.8518 |  | 1.06 | 0.82-1.37 | 0.6641 |  | 1.18 | 0.95-1.46 | 0.1425 |
| ***Duration of diabetes (years)*** | 1.00 | 0.97-1.04 | 0.8009 |  | 1.01 | 0.98-1.04 | 0.4838 |  | 1.03 | 1.01-1.06 | 0.0057 |
| ***Duration of retinopathy (years)*** | 0.95 | 0.90-1.01 | 0.1263 |  | 0.99 | 0.95-1.03 | 0.5627 |  | 0.99 | 0.95-1.02 | 0.4770 |
| ***Complications*** | 1.29 | 1.03-1.61 | 0.0273 |  | 1.33 | 1.13-1.55 | 0.0004 |  | 1.16 | 1.01-1.34 | 0.0393 |
| ***Glucose lowering therapies*** | 1.26 | 0.99-1.61 | 0.0572 |  | 1.05 | 0.87-1.27 | 0.6412 |  | 1.11 | 0.94-1.32 | 0.1979 |
| ***Insulin prescription*** | 2.29 | 1.38-3.80 | 0.0013 |  | 1.82 | 1.29-2.58 | 0.0007 |  | 1.83 | 1.34-2.50 | 0.0002 |
|  |  |  |  |  |  |  |  |  |  |  |  |
|  | **Value** | **95% CI (lower)** | **95% CI (upper)** |  | **Value** | **95% CI (lower)** | **95% CI (upper)** |  | **Value** | **95% CI (lower)** | **95% CI (upper)** |
| ***n after matching*** | 1,500 |  |  |  | 1,790 |  |  |  | 2,018 |  |  |
| ***C-statistic*** | 0.7470 | 0.7457 | 0.7483 |  | 0.7036 | 0.7028 | 0.7045 |  | 0.6789 | 0.6783 | 0.6796 |

Reference groups for categorical covariates include: white (ethnicity); North East (region); normal weight (BMI); non-smoker (smoking status); 0 units (alcohol consumption); and no insulin prescription (insulin use). N/A indicates that the sample after propensity score matching did not contain observations for the covariate.

Table S12: Summary table of the sensitivity analysis using lower targets

| QOF indicator | Primary Analysis  Adjusted HR (95% CI) | Sensitivity Analyses  Adjusted HR (95% CI) |
| --- | --- | --- |
| HbA1c | 0.64 (0.55-0.74) [HbA1c<7.5%] | 0.65 (0.56-0.76) [HbA1c<7.0%]  0.75 (0.62-0.91) [HbA1c<6.5%] |
| BP | 0.83 (0.72-0.94) [BP<140/80 mmHg] | 0.77 (0.67-0.90) [BP<130/80 mmHg] |
| Cholesterol | 0.80 (0.66-0.96) [Chol<5 mmol/L] | 0.81 (0.71-0.93) [Chol<4 mmol/L] |

Table S13: Univariate hazard ratios (with corresponding 95% CIs and p-values) for risk of sight-threatening diabetic retinopathy by each covariate across alternative QOF exposure definitions (HbA1c<7.0%; BP<130/80 mmHg; and cholesterol<4 mmol/L), after 1:1 propensity score matching.

|  | **Exposure Definition** | | | | | | | | | | | | | | |
| --- | --- | --- | --- | --- | --- | --- | --- | --- | --- | --- | --- | --- | --- | --- | --- |
|  | **Achieve HbA1c<7.0%**  **QOF Target** | | |  | **Achieve HbA1c<6.5%**  **QOF Target** | | |  | **Achieve Blood Pressure<130/80 QOF Target** | | |  | **Achieve Cholesterol<4 mmol/L**  **QOF Target** | | |
|  | **Hazard Ratio** | **95% CI** | **p** |  | **Hazard Ratio** | **95% CI** | **p** |  | **Hazard Ratio** | **95% CI** | **p** |  | **Hazard Ratio** | **95% CI** | **p** |
| **Exposure** | 0.64 | 0.54-0.74 | <0.0001 |  | 0.75 | 0.61-0.90 | 0.0028 |  | 0.78 | 0.67-0.90 | 0.0007 |  | 0.83 | 0.73-0.95 | 0.0060 |
| **Age** | 0.99 | 0.99-1.00 | 0.0474 |  | 0.99 | 0.98-1.00 | 0.0419 |  | 0.99 | 0.98-0.99 | 0.0003 |  | 0.99 | 0.98-0.99 | <0.0001 |
| **Sex: Female** | 0.93 | 0.79-1.08 | 0.3255 |  | 0.95 | 0.78-1.15 | 0.5719 |  | 1.03 | 0.89-1.19 | 0.7337 |  | 1.03 | 0.90-1.18 | 0.6696 |
| **Ethnicity: Asian** | 1.76 | 1.37-2.25 | <0.0001 |  | 1.67 | 1.16-2.39 | 0.0054 |  | 1.75 | 1.39-2.19 | <0.0001 |  | 1.65 | 1.33-2.05 | <0.0001 |
| **Ethnicity: Black** | 1.32 | 0.84-2.06 | 0.2248 |  | 1.16 | 0.64-2.11 | 0.6238 |  | 1.60 | 1.05-2.45 | 0.0298 |  | 1.20 | 0.79-1.83 | 0.3960 |
| **Ethnicity: Mixed** | 1.11 | 0.50-2.48 | 0.7953 |  | 0.42 | 0.06-2.99 | 0.3869 |  | 1.60 | 0.83-3.09 | 0.1615 |  | 1.11 | 0.53-2.34 | 0.7811 |
| **Ethnicity: Other** | 1.08 | 0.58-2.02 | 0.8034 |  | 0.90 | 0.37-2.16 | 0.8055 |  | 0.90 | 0.45-1.80 | 0.7609 |  | 1.12 | 0.65-1.93 | 0.6931 |
| **IMD** | 1.00 | 0.99-1.02 | 0.5233 |  | 1.00 | 0.99-1.02 | 0.7619 |  | 0.99 | 0.98-1.00 | 0.0791 |  | 1.00 | 0.99-1.01 | 0.7998 |
| **North West** | 0.85 | 0.69-1.06 | 0.1439 |  | 0.97 | 0.75-1.26 | 0.8354 |  | 0.84 | 0.68-1.03 | 0.1001 |  | 0.91 | 0.76-1.08 | 0.2802 |
| **Yorkshire & Humber** | 1.23 | 0.86-1.74 | 0.2521 |  | 1.29 | 0.83-2.00 | 0.2577 |  | 1.27 | 0.91-1.77 | 0.1634 |  | 1.15 | 0.84-1.58 | 0.3729 |
| **East Midlands** | 1.28 | 0.81-2.02 | 0.2907 |  | 1.18 | 0.65-2.15 | 0.5844 |  | 1.46 | 0.96-2.23 | 0.0801 |  | 1.19 | 0.79-1.80 | 0.4152 |
| **West Midlands** | 1.03 | 0.82-1.30 | 0.7826 |  | 0.99 | 0.73-1.32 | 0.9256 |  | 0.93 | 0.74-1.17 | 0.5518 |  | 0.92 | 0.75-1.13 | 0.4115 |
| **East of England** | 0.89 | 0.68-1.15 | 0.3629 |  | 1.07 | 0.78-1.47 | 0.6847 |  | 1.04 | 0.82-1.31 | 0.7601 |  | 0.95 | 0.76-1.18 | 0.6573 |
| **South West** | 1.09 | 0.88-1.35 | 0.4064 |  | 0.95 | 0.71-1.26 | 0.7072 |  | 1.14 | 0.93-1.40 | 0.2045 |  | 1.11 | 0.92-1.33 | 0.2653 |
| **South Central** | 0.95 | 0.75-1.21 | 0.6750 |  | 0.87 | 0.64-1.18 | 0.3759 |  | 1.01 | 0.81-1.27 | 0.9035 |  | 1.00 | 0.82-1.23 | 0.9631 |
| **London** | 0.94 | 0.75-1.18 | 0.6101 |  | 1.00 | 0.76-1.32 | 0.9842 |  | 0.95 | 0.76-1.17 | 0.6096 |  | 0.94 | 0.77-1.14 | 0.5091 |
| **South East Coast** | 1.03 | 0.82-1.29 | 0.7940 |  | 1.07 | 0.80-1.41 | 0.6605 |  | 0.90 | 0.71-1.13 | 0.3549 |  | 1.01 | 0.83-1.23 | 0.9495 |
| **BMI: Underweight** | 1.05 | 0.85-1.29 | 0.6763 |  | 0.88 | 0.68-1.14 | 0.3497 |  | 0.90 | 0.74-1.11 | 0.3300 |  | 0.90 | 0.75-1.09 | 0.2935 |
| **BMI: Overweight** | 1.16 | 0.99-1.35 | 0.0629 |  | 1.21 | 1.00-1.47 | 0.0550 |  | 1.02 | 0.87-1.18 | 0.8373 |  | 1.02 | 0.89-1.17 | 0.7714 |
| **BMI: Obese** | 0.84 | 0.72-0.98 | 0.0222 |  | 0.89 | 0.73-1.08 | 0.2325 |  | 1.04 | 0.90-1.20 | 0.6328 |  | 1.02 | 0.89-1.16 | 0.7806 |
| **BMI: Missing** | 1.84 | 0.95-3.55 | 0.0691 |  | 1.87 | 0.77-4.51 | 0.1652 |  | 1.92 | 0.96-3.86 | 0.0663 |  | 1.92 | 1.08-3.39 | 0.0251 |
| **Ex-Smoker** | 0.86 | 0.73-1.00 | 0.0544 |  | 0.89 | 0.73-1.08 | 0.2372 |  | 0.87 | 0.74-1.01 | 0.0609 |  | 0.86 | 0.75-0.99 | 0.0372 |
| **Current Smoker** | 0.91 | 0.72-1.15 | 0.4356 |  | 0.79 | 0.57-1.09 | 0.1590 |  | 0.89 | 0.71-1.11 | 0.3109 |  | 0.95 | 0.77-1.17 | 0.6264 |
| **Smoking: Missing** | 0.64 | 0.16-2.55 | 0.5248 |  | N/A | N/A | N/A |  | 1.33 | 0.50-3.56 | 0.5662 |  | 1.05 | 0.44-2.53 | 0.9112 |
| **Alcohol: 1-14** | 0.79 | 0.68-0.92 | 0.0027 |  | 0.96 | 0.79-1.16 | 0.6565 |  | 0.84 | 0.72-0.97 | 0.0200 |  | 0.86 | 0.75-0.98 | 0.0236 |
| **Alcohol: 15-42** | 1.21 | 0.96-1.53 | 0.1028 |  | 0.87 | 0.63-1.19 | 0.3847 |  | 0.91 | 0.70-1.19 | 0.4990 |  | 1.03 | 0.83-1.28 | 0.7737 |
| **Alcohol: >42** | 0.94 | 0.53-1.66 | 0.8189 |  | 0.70 | 0.33-1.48 | 0.3540 |  | 0.82 | 0.45-1.48 | 0.5091 |  | 0.65 | 0.36-1.18 | 0.1593 |
| **Alcohol: Missing** | 1.14 | 0.91-1.42 | 0.2597 |  | 1.28 | 0.96-1.70 | 0.0952 |  | 1.28 | 1.04-1.57 | 0.0174 |  | 1.18 | 0.98-1.44 | 0.0866 |
| **Morbidities** | 0.97 | 0.92-1.01 | 0.1638 |  | 0.96 | 0.90-1.02 | 0.1521 |  | 0.98 | 0.94-1.03 | 0.4393 |  | 0.97 | 0.93-1.01 | 0.1791 |
| **Prescriptions** | 1.01 | 1.01-1.02 | 0.0002 |  | 1.02 | 1.01-1.03 | 0.0001 |  | 1.02 | 1.01-1.02 | <0.0001 |  | 1.02 | 1.01-1.02 | <0.0001 |
| **Hospitalisations** | 1.19 | 1.06-1.33 | 0.0035 |  | 1.23 | 1.09-1.40 | 0.0013 |  | 1.21 | 1.09-1.35 | 0.0003 |  | 1.17 | 1.06-1.30 | 0.0028 |
| **Duration of diabetes (years)** | 1.02 | 1.01-1.03 | 0.0032 |  | 1.02 | 1.01-1.04 | 0.0017 |  | 1.02 | 1.00-1.03 | 0.0089 |  | 1.01 | 1.00-1.02 | 0.0103 |
| **Duration of retinopathy (years)** | 1.01 | 0.99-1.03 | 0.3823 |  | 1.01 | 0.98-1.03 | 0.6601 |  | 1.00 | 0.98-1.02 | 0.6939 |  | 1.00 | 0.99-1.02 | 0.7633 |
| **Complications** | 1.16 | 1.09-1.23 | <0.0001 |  | 1.19 | 1.10-1.28 | <0.0001 |  | 1.15 | 1.08-1.22 | <0.0001 |  | 1.13 | 1.08-1.20 | <0.0001 |
| **Glucose lowering therapies** | 1.36 | 1.26-1.48 | <0.0001 |  | 1.39 | 1.27-1.53 | <0.0001 |  | 1.43 | 1.33-1.53 | <0.0001 |  | 1.40 | 1.32-1.49 | <0.0001 |
| **Insulin prescription** | 2.22 | 1.87-2.64 | <0.0001 |  | 2.69 | 2.14-3.38 | <0.0001 |  | 2.51 | 2.16-2.92 | <0.0001 |  | 2.32 | 2.02-2.66 | <0.0001 |

Study sizes across exposures after 1:1 propensity score matching are found in Supplementary Table S13, as they are the same between univariate and multivariate analyses.

Table S14: Multivariate hazard ratios (with corresponding 95% CIs and p-values) for risk of sight-threatening diabetic retinopathy by each covariate across alternative QOF exposure definitions (HbA1c<7.0%; BP<130/80 mmHg; and cholesterol<4 mmol/L), after 1:1 propensity score matching.

|  | **Exposure Definition** | | | | | | | | | | | | | | |
| --- | --- | --- | --- | --- | --- | --- | --- | --- | --- | --- | --- | --- | --- | --- | --- |
|  | **Achieve HbA1c<7.0%**  **QOF Target** | | |  | **Achieve HbA1c<6.5%**  **QOF Target** | | |  | **Achieve Blood Pressure<130/80 QOF Target** | | |  | **Achieve Cholesterol<4 mmol/L**  **QOF Target** | | |
|  | **Hazard Ratio** | **95% CI** | **p** |  | **Hazard Ratio** | **95% CI** | **p** |  | **Hazard Ratio** | **95% CI** | **p** |  | **Hazard Ratio** | **95% CI** | **p** |
| **Exposure** | 0.65 | 0.56-0.76 | <0.0001 |  | 0.75 | 0.62-0.91 | 0.0036 |  | 0.77 | 0.67-0.90 | 0.0006 |  | 0.81 | 0.71-0.93 | 0.0020 |
| **Age** | 0.99 | 0.99-1.00 | 0.0712 |  | 0.99 | 0.98-1.00 | 0.0317 |  | 0.99 | 0.98-1.00 | 0.0242 |  | 0.99 | 0.98-1.00 | 0.0056 |
| **Sex: Female** | 1.11 | 0.93-1.32 | 0.2453 |  | 1.14 | 0.92-1.43 | 0.2312 |  | 1.19 | 1.00-1.40 | 0.0443 |  | 1.15 | 0.99-1.34 | 0.0739 |
| **Ethnicity: Asian** | 1.47 | 1.12-1.92 | 0.0057 |  | 1.23 | 0.83-1.81 | 0.2971 |  | 1.42 | 1.11-1.83 | 0.0062 |  | 1.32 | 1.04-1.68 | 0.0208 |
| **Ethnicity: Black** | 1.25 | 0.80-1.96 | 0.3315 |  | 0.93 | 0.51-1.71 | 0.8239 |  | 1.49 | 0.97-2.30 | 0.0680 |  | 1.07 | 0.70-1.65 | 0.7412 |
| **Ethnicity: Mixed** | 1.07 | 0.48-2.40 | 0.8682 |  | 0.36 | 0.05-2.57 | 0.3083 |  | 1.71 | 0.88-3.31 | 0.1135 |  | 1.12 | 0.53-2.35 | 0.7739 |
| **Ethnicity: Other** | 1.00 | 0.53-1.89 | 0.9933 |  | 0.77 | 0.32-1.89 | 0.5734 |  | 0.89 | 0.44-1.80 | 0.7416 |  | 1.11 | 0.64-1.94 | 0.7068 |
| **IMD** | 1.00 | 0.99-1.02 | 0.5865 |  | 1.00 | 0.98-1.02 | 0.7824 |  | 0.99 | 0.97-1.00 | 0.0806 |  | 1.00 | 0.99-1.01 | 0.7250 |
| **North West** | 0.62 | 0.40-0.95 | 0.0290 |  | 1.10 | 0.58-2.07 | 0.7738 |  | 0.71 | 0.45-1.12 | 0.1456 |  | 0.67 | 0.46-0.98 | 0.0397 |
| **Yorkshire & Humber** | 0.91 | 0.54-1.52 | 0.7100 |  | 1.55 | 0.75-3.24 | 0.2376 |  | 1.03 | 0.61-1.74 | 0.9099 |  | 0.84 | 0.53-1.33 | 0.4566 |
| **East Midlands** | 0.88 | 0.48-1.59 | 0.6659 |  | 1.31 | 0.56-3.03 | 0.5321 |  | 1.10 | 0.61-1.98 | 0.7464 |  | 0.84 | 0.49-1.44 | 0.5278 |
| **West Midlands** | 0.75 | 0.48-1.17 | 0.1991 |  | 1.14 | 0.59-2.19 | 0.7023 |  | 0.75 | 0.47-1.19 | 0.2236 |  | 0.67 | 0.45-1.00 | 0.0508 |
| **East of England** | 0.66 | 0.42-1.05 | 0.0818 |  | 1.23 | 0.63-2.41 | 0.5387 |  | 0.80 | 0.50-1.29 | 0.3659 |  | 0.70 | 0.47-1.05 | 0.0878 |
| **South West** | 0.75 | 0.49-1.16 | 0.1963 |  | 1.07 | 0.56-2.05 | 0.8378 |  | 0.88 | 0.56-1.38 | 0.5692 |  | 0.78 | 0.53-1.15 | 0.2148 |
| **South Central** | 0.68 | 0.43-1.06 | 0.0889 |  | 1.00 | 0.51-1.95 | 0.9935 |  | 0.78 | 0.49-1.24 | 0.2998 |  | 0.73 | 0.49-1.09 | 0.1218 |
| **London** | 0.65 | 0.42-1.02 | 0.0584 |  | 1.12 | 0.59-2.13 | 0.7363 |  | 0.76 | 0.48-1.21 | 0.2492 |  | 0.67 | 0.46-0.99 | 0.0457 |
| **South East Coast** | 0.73 | 0.47-1.13 | 0.1602 |  | 1.18 | 0.61-2.26 | 0.6242 |  | 0.69 | 0.43-1.10 | 0.1164 |  | 0.72 | 0.49-1.07 | 0.1056 |
| **BMI: Underweight** | 1.20 | 0.44-3.28 | 0.7157 |  | 1.56 | 0.38-6.36 | 0.5383 |  | 2.12 | 0.52-8.58 | 0.2943 |  | 1.63 | 0.52-5.13 | 0.4028 |
| **BMI: Overweight** | 1.25 | 0.46-3.36 | 0.6617 |  | 1.83 | 0.45-7.40 | 0.3951 |  | 2.26 | 0.56-9.08 | 0.2522 |  | 1.74 | 0.56-5.42 | 0.3419 |
| **BMI: Obese** | 0.97 | 0.36-2.62 | 0.9559 |  | 1.36 | 0.33-5.49 | 0.6697 |  | 1.99 | 0.49-8.00 | 0.3347 |  | 1.51 | 0.48-4.71 | 0.4800 |
| **BMI: Missing** | 2.10 | 0.64-6.83 | 0.2201 |  | 2.71 | 0.52-14.05 | 0.2359 |  | 3.68 | 0.78-17.42 | 0.1000 |  | 3.09 | 0.87-10.98 | 0.0814 |
| **Ex-Smoker** | 0.86 | 0.72-1.02 | 0.0848 |  | 0.85 | 0.69-1.06 | 0.1509 |  | 0.88 | 0.75-1.04 | 0.1390 |  | 0.88 | 0.76-1.02 | 0.0911 |
| **Current Smoker** | 0.84 | 0.66-1.08 | 0.1845 |  | 0.71 | 0.50-0.99 | 0.0445 |  | 0.84 | 0.66-1.07 | 0.1517 |  | 0.86 | 0.69-1.07 | 0.1896 |
| **Smoking: Missing** | 0.80 | 0.20-3.23 | 0.7555 |  | N/A | N/A | N/A |  | 1.39 | 0.52-3.75 | 0.5153 |  | 1.13 | 0.46-2.73 | 0.7932 |
| **Alcohol: 1-14** | 0.86 | 0.70-1.05 | 0.1373 |  | 0.98 | 0.75-1.29 | 0.8893 |  | 0.89 | 0.73-1.09 | 0.2743 |  | 0.89 | 0.75-1.07 | 0.2144 |
| **Alcohol: 15-42** | 1.13 | 0.84-1.52 | 0.4032 |  | 0.93 | 0.62-1.39 | 0.7170 |  | 0.95 | 0.69-1.30 | 0.7450 |  | 1.05 | 0.80-1.37 | 0.7471 |
| **Alcohol: >42** | 0.97 | 0.53-1.77 | 0.9297 |  | 0.77 | 0.35-1.69 | 0.5071 |  | 0.84 | 0.45-1.57 | 0.5884 |  | 0.67 | 0.36-1.25 | 0.2087 |
| **Alcohol: Missing** | 0.94 | 0.72-1.24 | 0.6798 |  | 1.09 | 0.76-1.56 | 0.6326 |  | 0.98 | 0.76-1.26 | 0.8520 |  | 0.95 | 0.75-1.20 | 0.6610 |
| **Morbidities** | 0.90 | 0.85-0.96 | 0.0008 |  | 0.88 | 0.81-0.95 | 0.0006 |  | 0.93 | 0.87-0.98 | 0.0075 |  | 0.92 | 0.88-0.97 | 0.0020 |
| **Prescriptions** | 1.00 | 0.99-1.01 | 0.6800 |  | 1.00 | 0.98-1.01 | 0.8309 |  | 1.00 | 0.99-1.01 | 0.3436 |  | 1.00 | 0.99-1.01 | 0.4090 |
| **Hospitalisations** | 1.16 | 1.02-1.30 | 0.0184 |  | 1.20 | 1.05-1.37 | 0.0070 |  | 1.19 | 1.06-1.33 | 0.0023 |  | 1.15 | 1.04-1.29 | 0.0087 |
| **Duration of diabetes (years)** | 1.01 | 1.00-1.02 | 0.1060 |  | 1.02 | 1.00-1.03 | 0.0271 |  | 1.01 | 1.00-1.02 | 0.2160 |  | 1.01 | 1.00-1.02 | 0.2372 |
| **Duration of retinopathy (years)** | 1.01 | 0.99-1.03 | 0.3829 |  | 1.00 | 0.98-1.03 | 0.7926 |  | 0.99 | 0.97-1.01 | 0.5219 |  | 1.00 | 0.98-1.02 | 0.9134 |
| **Complications** | 1.23 | 1.14-1.33 | <0.0001 |  | 1.27 | 1.15-1.41 | <0.0001 |  | 1.20 | 1.11-1.29 | <0.0001 |  | 1.19 | 1.12-1.28 | <0.0001 |
| **Glucose lowering therapies** | 1.26 | 1.14-1.39 | <0.0001 |  | 1.24 | 1.10-1.40 | 0.0007 |  | 1.29 | 1.18-1.41 | <0.0001 |  | 1.28 | 1.18-1.38 | <0.0001 |
| **Insulin prescription** | 1.78 | 1.47-2.15 | <0.0001 |  | 1.88 | 1.45-2.45 | <0.0001 |  | 1.92 | 1.61-2.27 | <0.0001 |  | 1.80 | 1.55-2.11 | <0.0001 |
|  |  |  |  |  |  |  |  |  |  |  |  |  |  |  |  |
|  | **Value** | **95% CI (lower)** | **95% CI (upper)** |  | **Value** | **95% CI (lower)** | **95% CI (upper)** |  | **Value** | **95% CI (lower)** | **95% CI (upper)** |  | **Value** | **95% CI (lower)** | **95% CI (upper)** |
| **n after matching** | 13,618 |  |  |  | 10,336 |  |  |  | 13,254 |  |  |  | 16,214 |  |  |
| **C-statistic** | 0.6606 | 0.6604 | 0.6609 |  | 0.6724 | 0.6720 | 0.6728 |  | 0.6819 | 0.6817 | 0.6821 |  | 0.6649 | 0.6647 | 0.6651 |

Reference groups for categorical covariates include: white (ethnicity); North East (region); normal weight (BMI); non-smoker (smoking status); 0 units (alcohol consumption); and no insulin prescription (insulin use).

Table S15: Univariate hazard ratios (with corresponding 95% CIs and p-values) for risk of sight-threatening diabetic retinopathy by each covariate for those who meet exactly 2 QOF targets versus those who achieve less than 2 QOF targets, after 1:1 propensity score matching.

|  | **Exposure Definition** | | | |
| --- | --- | --- | --- | --- |
|  |  | **Achieve any 2 QOF Targets (vs. <2)** | | |
|  |  | **Hazard Ratio** | **95% CI** | **p** |
| **Exposure** |  | 0.63 | 0.54-0.73 | <0.0001 |
| **Age** |  | 0.98 | 0.98-0.99 | <0.0001 |
| **Sex: Female** |  | 1.01 | 0.87-1.18 | 0.8623 |
| **Ethnicity: Asian** |  | 1.55 | 1.20-2.00 | 0.0007 |
| **Ethnicity: Black** |  | 1.51 | 1.01-2.26 | 0.0425 |
| **Ethnicity: Mixed** |  | 1.91 | 0.95-3.84 | 0.0683 |
| **Ethnicity: Other** |  | 1.05 | 0.56-1.96 | 0.8764 |
| **IMD** |  | 1.00 | 0.98-1.01 | 0.7138 |
| **North West** |  | 0.95 | 0.77-1.17 | 0.6133 |
| **Yorkshire & Humber** |  | 0.85 | 0.55-1.32 | 0.4716 |
| **East Midlands** |  | 1.38 | 0.87-2.17 | 0.1710 |
| **West Midlands** |  | 1.02 | 0.80-1.28 | 0.9000 |
| **East of England** |  | 1.00 | 0.78-1.29 | 0.9874 |
| **South West** |  | 1.01 | 0.81-1.26 | 0.9120 |
| **South Central** |  | 0.97 | 0.76-1.23 | 0.7735 |
| **London** |  | 0.88 | 0.70-1.11 | 0.2982 |
| **South East Coast** |  | 1.01 | 0.80-1.27 | 0.9412 |
| **BMI: Underweight** |  | 0.89 | 0.70-1.13 | 0.3405 |
| **BMI: Overweight** |  | 1.01 | 0.86-1.19 | 0.8976 |
| **BMI: Obese** |  | 1.03 | 0.88-1.20 | 0.7057 |
| **BMI: Missing** |  | 1.77 | 0.91-3.41 | 0.0905 |
| **Ex-Smoker** |  | 0.82 | 0.69-0.96 | 0.0152 |
| **Current Smoker** |  | 0.97 | 0.77-1.22 | 0.7931 |
| **Smoking: Missing** |  | 1.30 | 0.49-3.48 | 0.6005 |
| **Alcohol: 1-14** |  | 0.91 | 0.78-1.07 | 0.2535 |
| **Alcohol: 15-42** |  | 0.94 | 0.72-1.23 | 0.6481 |
| **Alcohol: >42** |  | 0.60 | 0.30-1.20 | 0.1477 |
| **Alcohol: Missing** |  | 1.16 | 0.94-1.44 | 0.1636 |
| **Morbidities** |  | 0.95 | 0.90-1.00 | 0.0332 |
| **Prescriptions** |  | 1.01 | 1.01-1.02 | 0.0002 |
| **Hospitalisations** |  | 1.11 | 0.98-1.25 | 0.1000 |
| **Duration of diabetes (years)** |  | 1.01 | 1.00-1.03 | 0.0376 |
| **Duration of retinopathy (years)** |  | 1.00 | 0.98-1.02 | 0.6751 |
| **Complications** |  | 1.12 | 1.05-1.19 | 0.0006 |
| **Glucose lowering therapies** |  | 1.39 | 1.30-1.49 | <0.0001 |
| **Insulin prescription** |  | 2.30 | 1.97-2.68 | <0.0001 |

Study sizes across exposures after 1:1 propensity score matching are found in Supplementary Table S15, as they are the same between univariate and multivariate analyses.

Table S16: Multivariate hazard ratios (with corresponding 95% CIs and p-values) for risk of sight-threatening diabetic retinopathy by each covariate for those who achieve exactly 2 QOF targets versus those who achieve fewer than 2 QOF targets, after 1:1 propensity score matching.

|  | **Exposure Definition** | | | |
| --- | --- | --- | --- | --- |
|  |  | **Achieve any 2 QOF Targets (vs. <2)** | | |
|  |  | **Hazard Ratio** | **95% CI** | **p** |
| **Exposure** |  | 0.67 | 0.57-0.78 | <0.0001 |
| **Age** |  | 0.98 | 0.98-0.99 | <0.0001 |
| **Sex: Female** |  | 1.22 | 1.02-1.45 | 0.0289 |
| **Ethnicity: Asian** |  | 1.23 | 0.93-1.63 | 0.1508 |
| **Ethnicity: Black** |  | 1.30 | 0.87-1.96 | 0.2030 |
| **Ethnicity: Mixed** |  | 1.86 | 0.92-3.76 | 0.0835 |
| **Ethnicity: Other** |  | 1.02 | 0.54-1.92 | 0.9519 |
| **IMD** |  | 1.00 | 0.98-1.01 | 0.6307 |
| **North West** |  | 0.60 | 0.40-0.91 | 0.0150 |
| **Yorkshire & Humber** |  | 0.53 | 0.30-0.93 | 0.0264 |
| **East Midlands** |  | 0.83 | 0.46-1.47 | 0.5185 |
| **West Midlands** |  | 0.63 | 0.41-0.96 | 0.0329 |
| **East of England** |  | 0.63 | 0.41-0.98 | 0.0385 |
| **South West** |  | 0.62 | 0.41-0.94 | 0.0238 |
| **South Central** |  | 0.61 | 0.40-0.94 | 0.0237 |
| **London** |  | 0.56 | 0.37-0.85 | 0.0063 |
| **South East Coast** |  | 0.61 | 0.40-0.93 | 0.0226 |
| **BMI: Underweight** |  | 1.67 | 0.41-6.82 | 0.4747 |
| **BMI: Overweight** |  | 1.73 | 0.43-6.96 | 0.4437 |
| **BMI: Obese** |  | 1.49 | 0.37-6.00 | 0.5755 |
| **BMI: Missing** |  | 2.93 | 0.63-13.64 | 0.1705 |
| **Ex-Smoker** |  | 0.85 | 0.71-1.01 | 0.0700 |
| **Current Smoker** |  | 0.86 | 0.68-1.10 | 0.2390 |
| **Smoking: Missing** |  | 1.52 | 0.56-4.09 | 0.4094 |
| **Alcohol: 1-14** |  | 0.96 | 0.77-1.18 | 0.6745 |
| **Alcohol: 15-42** |  | 1.01 | 0.72-1.40 | 0.9673 |
| **Alcohol: >42** |  | 0.60 | 0.29-1.23 | 0.1632 |
| **Alcohol: Missing** |  | 0.96 | 0.73-1.26 | 0.7797 |
| **Morbidities** |  | 0.92 | 0.86-0.97 | 0.0042 |
| **Prescriptions** |  | 0.99 | 0.98-1.00 | 0.0541 |
| **Hospitalisations** |  | 1.10 | 0.97-1.25 | 0.1279 |
| **Duration of diabetes (years)** |  | 1.01 | 0.99-1.02 | 0.2349 |
| **Duration of retinopathy (years)** |  | 1.00 | 0.98-1.02 | 0.7334 |
| **Complications** |  | 1.22 | 1.13-1.32 | <0.0001 |
| **Glucose lowering therapies** |  | 1.28 | 1.17-1.40 | <0.0001 |
| **Insulin prescription** |  | 1.83 | 1.54-2.18 | <0.0001 |
|  |  |  |  |  |
|  |  | **Value** | **95% CI (lower)** | **95% CI (upper)** |
| **n after matching** |  | 10,072 |  |  |
| **C-statistic** |  | 0.6815 | 0.6813 | 0.6817 |

Reference groups for categorical covariates include: white (ethnicity); North East (region); normal weight (BMI); non-smoker (smoking status); 0 units (alcohol consumption); and no insulin prescription (insulin use).

Figure S1: Flow diagram showing the derivation of the study cohort

Figure S2: Kaplan-Meier survival curves (and corresponding 95% CIs) for risk of sight-threatening diabetic retinopathy after 1:1 propensity score matching across QOF exposure definitions, *among those who meet all other QOF targets*.

| Exposure: HbA1c QOF Target  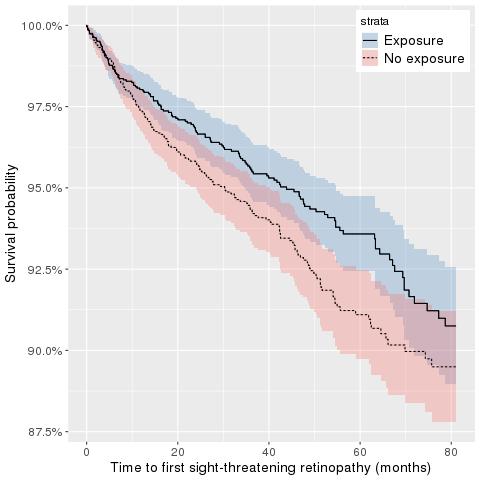 | Exposure: BP QOF Target  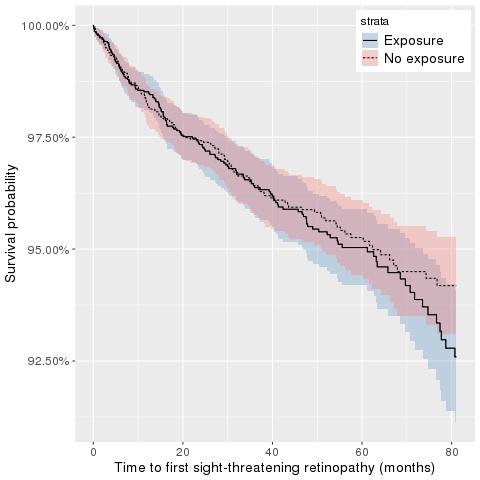 | Exposure: Cholesterol QOF Target  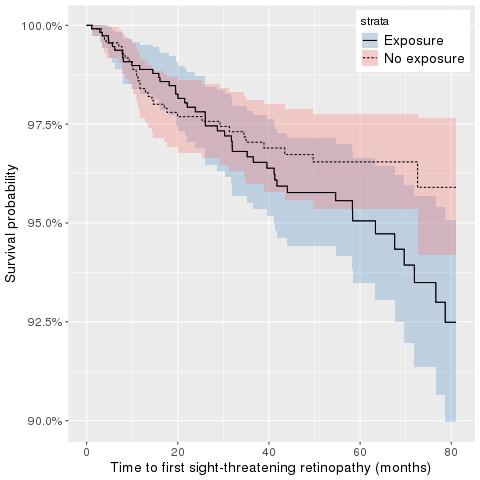 |
| --- | --- | --- |

Figure S3: Kaplan-Meier survival curves (and corresponding 95% CIs) for risk of sight-threatening diabetic retinopathy after 1:1 propensity score matching across QOF exposure definitions, *among those who meet none of the other QOF targets*.

| Exposure: HbA1c QOF Target  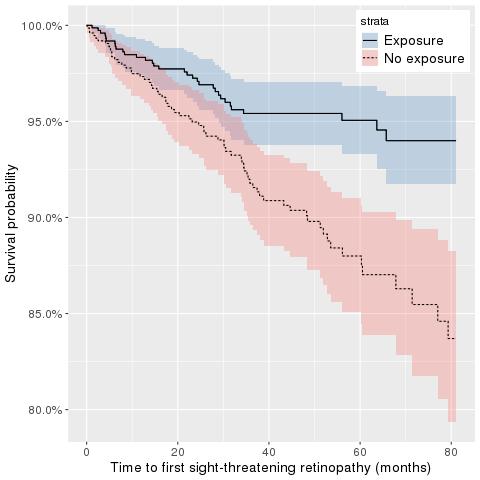 | Exposure: BP QOF Target  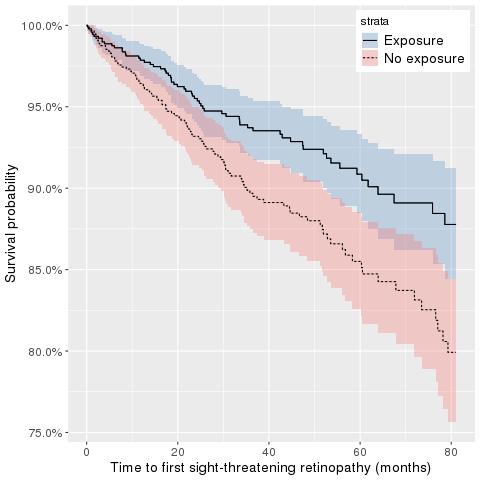 | Exposure: Cholesterol QOF Target  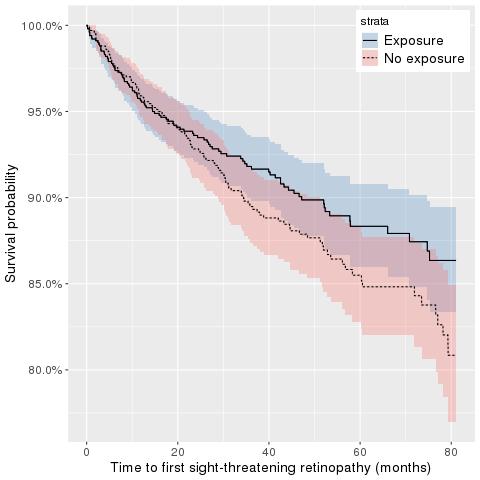 |
| --- | --- | --- |

Figure S4: Kaplan-Meier survival curves (and corresponding 95% CIs) for risk of sight-threatening diabetic retinopathy after 1:1 propensity score matching across alternative QOF exposure definitions (HbA1c<7.0%; BP<130/80 mmHg; and cholesterol<4 mmol/L).

| Exposure: HbA1c<7.0%  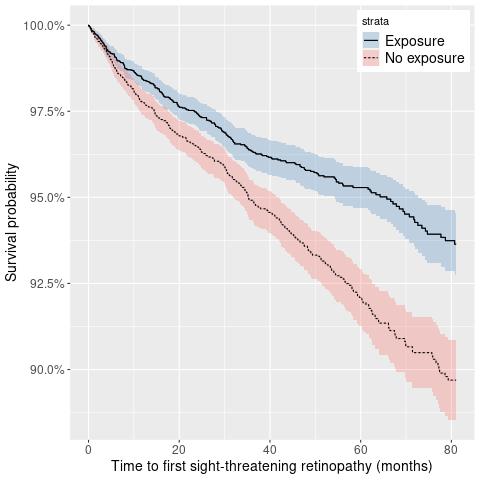 | Exposure: HbA1c<6.5%  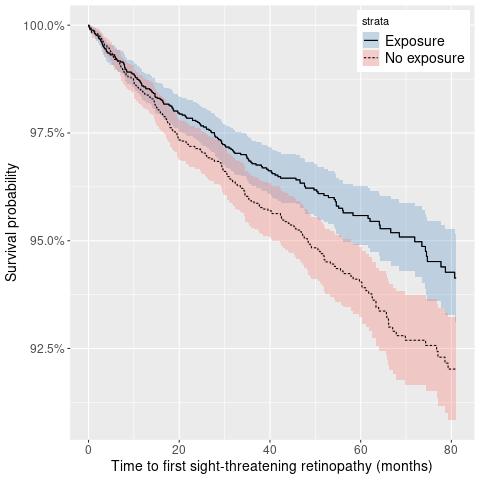 |
| --- | --- |
| Exposure: BP<130/80 mmHg  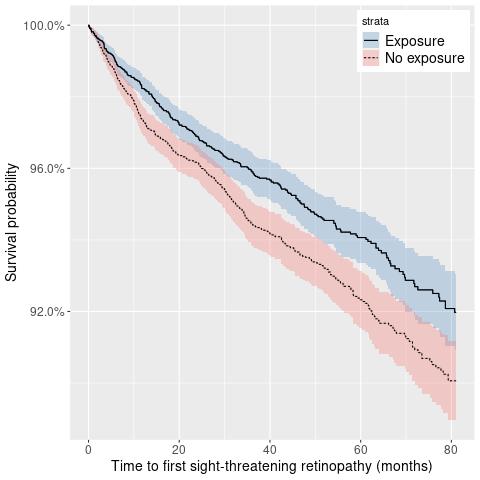 | Exposure: Chol<4mmol/L  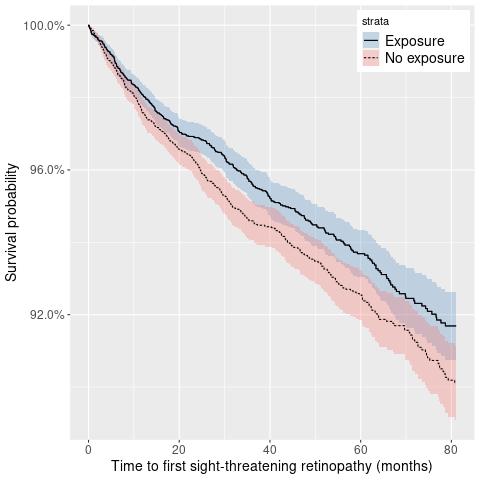 |

Figure S5: Kaplan-Meier survival curves (and corresponding 95% CIs) for risk of sight-threatening diabetic retinopathy for those who achieve exactly 2 QOF targets versus those who achieve fewer than 2 QOF targets, after 1:1 propensity score matching.

| Exposure: 2 QOF Targets (vs. <2)  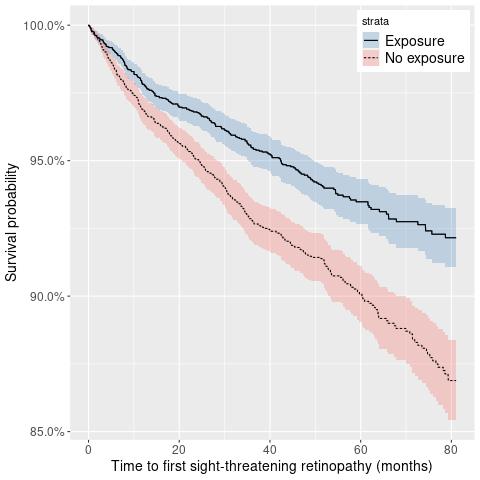 |
| --- |
